# Supplementary material for: Electrothermal mineralization of per- and polyfluoroalkyl substances for soil remediation
Source: Nat Commun. 2024 Jul 20;15:6117. doi: 10.1038/s41467-024-49809-6 (PMC11271446; doi:10.1038/s41467-024-49809-6)
Supplement: Supplementary file 1 — Supplementary Information [file 41467_2024_49809_MOESM1_ESM.pdf]

Supplementary Information

Electrothermal mineralization of per- and polyfluoroalkyl substances for soil remediation

Yi Cheng, Bing Deng, Phelecia Scotland, Lucas Eddy, Arman Hassan, Bo Wang, Karla J. Silva, Bowen Li, Kevin M. Wyss, Mine G. Ucak-Astarlioglu, Jinhang Chen, Qiming Liu, Tengda Si, Shichen Xu, Xiaodong Gao, Khalil JeBailey, Debadrita Jana, Mark Albert Torres, Michael S. Wong, Boris I. Yakobson, Christopher Griggs, Matthew A. McCary, Yufeng Zhao, and James M. Tour

The supplementary information includes:

Supplementary Note 1-4

Supplementary Figs. 1-66

Supplementary Tables 1-17

Supplementary References

Supplementary Note 1. Strategy for scaling up the REM process.

REM process is promising to realize the scalable remediation of PFAS-contaminated soil. Here, we first analyzed the theoretical feasibility of scaling up and then demonstrated the upscaling of the process by treating tens-of-gram-scale samples in a batch.

1.1 Scaling rule of REM process by theoretical analysis. For the REM process, the PFAS mineralization in the soil mostly depends on the treating temperature and the time (Fig. 1d and Fig. 2a-c). Hence, the REM temperature across the soil sample is critical when scaling up the process. For REM process, the input heat (Q) can be calculated by equation (S1),

$$Q = I^2 R t \tag{S1}$$

where I is the current passing through the sample, R is the sample resistance, and t is the discharging time. The heat amount per volume (Q_v) is calculated by equation (S2),

$$Q_\mathrm{v} = j^2 \rho_\mathrm{e} t \tag{S2}$$

where j is the current density, and ρ_e is the electrical resistivity. For a specific sample, the electrical resistivity (ρ_e) is constant.

The temperature difference (ΔT) is proportional to the heat amount by equation (S3),

$$Q = C_\mathrm{p} m \Delta T \tag{S3}$$

where C_p is heat capacity and m is the mass of the sample. The equation (S3) could be reformulated per volume to equation (S4),

$$Q_\mathrm{v} = C_\mathrm{p} \rho_\mathrm{m} \Delta T \tag{S4}$$

where ρ_m is the density of the sample. For a specific sample, the C_p and ρ_m were constant; hence, maintaining a constant Q_v is proportional to ΔT.

The charge amount (q) in the capacitor bank could be calculated by equation (S5),

$$q = CV \tag{S5}$$

where C is the capacitance of the capacitor bank, and V is the voltage of the capacitor bank. Assuming that all the charges in the capacitor bank are discharged within the time of t, the current density (j) could be calculated by equation (S6),

$$j = \frac{I}{S} = \frac{CV}{St} \tag{S6}$$

where S is the cross-sectional area of the sample. Since we usually use the cylinder-shaped sample, the mass (m) could be calculated by equation (S7),

$$m = \rho_{\mathrm{m}} S L \tag{S7}$$

where ρ_{m} is the density of the sample, S is the cross-sectional area of the sample, and L is the length of the sample. For a specific type of sample, such as soil and biochar, the density (ρ_{m}) is the same.

To summarize, we can obtain the below equation (S8) that determines the current density,

$$j = \frac{CV\rho_{\mathrm{m}}L}{mt} \tag{S8}$$

Hence, ΔT can be calculated following the equation (S9) below.

$$\Delta T = \frac{C^2V^2L^2\rho_{\mathrm{m}}\rho_{\mathrm{e}}}{m^2tC_{\mathrm{p}}} \tag{S9}$$

As aforementioned, to scale up the REM process, increasing the mass (m) of the sample is required, while a constant temperature difference (ΔT) should be maintained. Two routes can be adopted: (1) increasing the input voltage (V), and/or (2) increasing the capacitance (C) of the REM system.

1.2 Scaling up to gram scale per batch. In our first-generation REM setup, we used a capacitor bank composed of 10 aluminum electrolytic capacitors (450 V, 6 mF, Mouser #80-PEH200YX460BQU2) with a total capacitance of C_0 = 0.06 F (Supplementary Fig. 1). In a small-scale experiment, the input voltage (V_0) of 100 V and capacitance (C_0) of 0.06 F were used for the sample mass of m_0 = 0.30 g. Here, we demonstrated the scaling up of the REM process to a mass

of m_1 = 10 g based on a second-generation setup with larger capacitance of C_1 = 0.624 F (Supplementary Fig. 54). Thus, we can obtain the below formula of equation (S10),

$$\frac{m_1}{m_0} = \frac{C_1 V_1}{C_0 V_0} \quad \text{(S10)}$$

For the mass of m_1 = 11 g and C_1 = 0.624 F, we used a REM voltage of $V_1 \approx$ 300 V, which basically fits with the eq 10. The peak temperature during this REM process is ~1600 °C (Supplementary Fig. 54e), similar to small-batch temperature (Fig. 1c), proving the efficiency of the scale-up.

Supplementary Note 2. Electric field simulation.

The simulation was conducted based on the finite element method using the COMSOL Multiphysics 5.5 software. The Electric Currents interface under the AC/DC module was used as the model. The geometric configuration and materials parameters are shown in Supplementary Table 10. The electrical conductivity of the mixed soil/carbon additive was calculated by measuring the resistance. Other material parameters are from the physical constant table. The geometric configurations and boundary conditions are shown in Supplementary Fig. 57. The simulated electric potential map (Supplementary Fig. 58a) shows the linear decrease of electric potential from the Electrical Potential electrodes to the ground electrodes. The simulated in-plane current density map and in-depth map are shown in Fig. 5c and Supplementary Fig. 58b. Both maps are uniform, proving the homogeneous heating capability of the REM process. Current density is critical for the Joule heating. According to $Q = I^2Rt$, since soil conductivity is the same, similar current density leads to a similar heating effect. In the kilogram-scale sample with 200 V input, the current density is ~1000 A m^{-2}.

To assess the practical applicability of the REM process, we further extended the simulation to a 1 m × 1 m × 1 m scale. The configuration is shown in Supplementary Fig. 59a,b. The material properties and boundary conditions are the same as in the aforementioned small-scale sample. In this case, the voltage input with 1000 V was applied (Supplementary Fig. 59c). The current density in the central position is calculated to be ~800 A m^{-2}, similar to that in the small-scale clay pot. According to our theoretical analysis (Supplementary Note 1), the current density determines the accessible temperature of the sample during the REM process. This indicates that a comparable temperature of ~1000 °C can be achieved under such a voltage input for the large-scale 1 m^3 sample.

To reveal the relationship of electrode surface area on the heating efficacy, we conducted a simulation on a 1 m × 1 m × 1 m volume sample with different electrode surface area (Supplementary Fig. 60). With the increase of each electrode surface area from 0.25 to 0.75 m^2, the current density at the center position increases from 730 to 870 A m^2 (Supplementary Fig. 61), which indicates a higher REM temperature since the current density determines the temperature according to our analysis (Supplementary Note 1). Therefore, during REM, a higher electrode surface area can facilitate a higher REM temperature and thus a higher PFOA removal efficiency.

Supplementary Note 3. Electrical energy consumption for REM process.

The energy consumption of the REM process is calculated using equation (S11),

$$E = \frac{(V_1^2 - V_2^2) \times C \times n}{2 \times M} \tag{S11}$$

Where E is the consumed energy per gram (kJ g^{-1}), V_1 and V_2 are the voltage before and after REM, respectively, C is the capacitance (C = 60 mF), n is the times of pulses, and M is the mass per batch.

For PFAS mineralization, V_1 = 100 V, V_2 = 0 V, M = 0.3 g (0.2 g of soil mixed with 0.1 g of conductive additive), and n = 1. Therefore, the energy consumption can be calculated as follows: E = 1.5 kJ g^{-1} = 420 kWh t^{-1}.

Given that the industrial price of electrical energy in Texas, USA is \$0.0587 kWh^{-1}, US Energy Information Administration, ref[1]), the cost for the remediation of 1 tonne soil can be estimated to be: P = \$16.8 t^{-1}.

The material cost mainly comes from the conductive additives. During our REM process, we mixed PFAS-contaminated soil and conductive additives (biochar or metcoke) with a mass ratio of 2:1 (1 tonne of soil requires 0.5 tonne of conductive additives).

For the biochar, after centrifugation, ~85 wt% biochar can be recycled and reused (Supplementary Fig. 19). Thus, the consumed biochar is 0.075 tonne for 1-tonne soil treatment. Considering the bulk price of biochar is ~\$1400 $tonne^{-1}$ (ref[2]), the material cost for REM treating PFAS-contaminated soil could be calculated as P(Material) = \$105.0 $tonne^{-1}$. Hence, when biochar is used as the conductive additive, the total cost would be P(total) = \$121.8 $tonne^{-1}$.

When using metcoke as the conductive additive, ~93 wt% metcoke can be recycled and reused after a simple sieving method (Supplementary Fig. 23). Thus, the consumed metcoke is 0.035 tonne for 1-tonne soil treatment. The bulk price of metcoke is ~\$150 $tonne^{-1}$ (ref[3]). Hence,

the material cost for the treatment of 1-tonne soil would be P(Material) = \$5.3 tonne^{-1}. Hence, when metcoke is used as the conductive additive, the total cost would be P(total) = \$22.1 tonne^{-1}.

Supplementary Note 4. Life Cycle Analysis.

4.1 Goal and scope.

This study follows the ISO 14044 (ref[4]) requirements with the aim of comparing the potential environmental impacts of the PFAS remediation processes, including thermal treatment, chemical oxidation, and ball milling, with the REM process. Two different conductive additives (biochar and metcoke) were considered in the REM process. The goal of the analysis is to determine whether FJH process can result in reduced energy consumption, GHG emissions, and water consumption.

4.2 Scenario description and system boundaries.

Five scenarios were considered in this study (Fig. 5d and Supplementary Fig. 63). In each scenario, 1 tonne of PFAS-contaminated soil is used as the baseline with the PFAS concentration of 100 ppm, and all other material flows are normalized to the treatment of 1 tonne of PFAS-contaminated soil (Supplementary Table 11). REM that can be conducted on-site (Fig. 1a). Other off-site scenarios require the excavation, transportation, and subsequent return of the soil during the remediation processes.

Scenario 1 Thermal treatment: In this scenario[5], PFAS-contaminated soil (1 tonne) was excavated from the site and transported off-site. After loading into a furnace by the conventional thermal treatment process, the remediated soil was transported back and returned to the original site. Because of the exposure to the environment during thermal treatment, PFAS would be degraded into perfluorinated gaseous species. Assuming a PFOA content of 100 ppm in the

contaminated soil and C_2F_4 as the main gaseous products, 0.09 kg of harmful perfluorinated gas would be emitted to the environment, when treating 1 tonne of soil.

Scenario 2 Chemical oxidation: In this scenario[6], PFAS-contaminated soil (1 tonne) was excavated from the site and transported off-site. Potassium permanganate (10 kg) was mixed with PFAS-contaminated soil (1 tonne). The mixture was then dispersed in water (10 tonnes) and then heated in the water bath. The remediated soil was then filtered, dried, and transported back and returned to the original site. Assuming a PFOA content of 100 ppm in the contaminated soil, when treating 1 tonne of the contaminated soil, based on the chemical equation: $32KMnO_4 + 5C_8F_{15}O_2H + 19H_2O \rightarrow 40CO_2 + 32KF + 32MnO_2 + 43HF$, 9.76 kg of potassium permanganate remained after the reaction and 0.04 kg HF would generate, which are regarded as the toxic chemical waste.

Scenario 3 Ball milling: In this scenario[7], PFAS-contaminated soil (1 tonne) was excavated from the site and transported off-site. Potassium hydroxide (9.5 kg) was mixed with PFAS-contaminated soil (1 tonne). The mixture was then milled for 3 hours and the remediated soil was transported back and returned to the original site. Assuming a PFOA content of 100 ppm in the contaminated soil and all F atoms in PFOA were mineralized by potassium ions in KF, 9.30 kg of potassium hydroxide remained after the reaction, which is regarded as the chemical waste.

Scenario 4 REM using biochar: In this scenario, biochar was used as the conductive additive. Commercial biochar (0.5 tonne) was pretreated by an electrothermal process and then mixed with contaminated soil (1 tonne). Afterwards, the mixture was treated by REM process and then the residual biochar in the mixture is separated by cyclone separation process (0.43 tonne).

Scenario 5 REM using metcoke: In this scenario, metcoke was used as the conductive additive. Mecoke (0.5 tonne) was mixed with contaminated soil (1 tonne). Afterwards, the mixture

was treated by REM process and then the metcoke in the mixture is separated by sieving (0.47 tonne).

4.3 Life cycle inventory.

The environmental impacts, including energy consumption demand, GHG emission and water consumption for the materials production, processing, are summarized in Supplementary Table 12. The values are explained below. Note that 1 MJ electricity produces 0.13 kg GHG and consumes 0.67 kg water from Argonne GREET model[8].

Materials production: The GHG emission, energy consumption and water consumption for biochar (5.08 kg tonne^{-1}, 200025 MJ tonne^{-1}, 4.70 kg tonne^{-1}), potassium permanganate (19480 kg tonne^{-1}, 1891 MJ tonne^{-1}, 4382 kg tonne^{-1}), and potassium hydroxide (1980 kg tonne^{-1}, 28000 MJ tonne^{-1}, 17221 kg tonne^{-1}) are from the Argonne GREET model[8].

Processing - Mixing: Energy input is needed for the mixing process, including the mixing of soil with oxidizing agent for chemical oxidation, the mixing of soil with base for ball milling, and the mixing of soil with conductive additives for REM. We assume that the mixing is conducted using an electrically driven Powder Mixer[9] with the energy consumption of 9.43 MJ tonne^{-1}. Correspondingly, 1.23 kg tonne^{-1} GHG emitted and 6.32 kg tonne^{-1} water consumed during the mixing process.

Processing - Evacuation: The soil evacuation was conducted using a hydraulic digger and skid steer loader. The estimated energy consumption was 4.53 MJ tonne^{-1} (ref[10]). Correspondingly, 0.59 kg tonne^{-1} GHG emitted and 3.04 kg tonne^{-1} water consumed during the process.

Processing – Transportation: The remediating site is estimated to be 200 km away from the contaminated site. Therefore, contaminated soil needs to be transported for remediation after evacuation and for refilling after remediation. For the transportation of 1 tonne of soil over a

distance of 200 km, energy consumption is 447 MJ, GHG emission is estimated to be 33.2 kg and corresponding water consumption is estimated to be 299.5 kg [11].

Processing – Furnace heating: Assuming the heating rate of 25 °C min^{-1} and the treating time of 75 minutes[5], the electrical energy consumption of a commercial furnace is estimated to be 3874 MJ $tonne^{-1}$ (ref[12]). Correspondingly, 504 kg $tonne^{-1}$ GHG emitted and 2595.6 kg $tonne^{-1}$ water consumed during the process.

Processing – Water bath heating: During chemical oxidation process, 1 tonne of contaminated soil dispersed in 10 tons of water needs to be immersed in a water bath. Considering the 18-day heating at 85 °C[6], the energy consumption is 1866 MJ $tonne^{-1}$ (ref[13]). Correspondingly, 243 kg $tonne^{-1}$ GHG emitted and 1260 kg $tonne^{-1}$ water consumed during the mixing process.

Processing – Filtration. We assume that the filtration is conducted using a filtration system. The energy consumption is estimated to be 2.2 MJ $tonne^{-1}$. Correspondingly, 0.29 kg $tonne^{-1}$ GHG emitted and 1.47 kg $tonne^{-1}$ water consumed during the filtration process.

Processing – Ball milling: The ball milling was conducted by a commercial ball milling machine with the energy consumption of 3456 MJ $tonne^{-1}$ after 3-hour ball milling[7]. Correspondingly, 449 kg $tonne^{-1}$ GHG and 2316 kg $tonne^{-1}$ water consumed emitted during the ball milling process[14].

Processing – Refilling: The remediated soil needs to be refilled back to the original site after remediation. The energy consumption is estimated to be 19.6 MJ ton^{-1} (ref[15]). Correspondingly, 2.55 kg $tonne^{-1}$ and 13.13 kg $tonne^{-1}$ water consumed GHG emitted during the process.

Processing – Biochar pretreatment: Biochar was pretreated by electrothermal process. The energy consumption can be calculated by equation (S11). Herein, V_1 = 60 V, V_2 = 0 V, M = 0.3 g, and n = 1. Therefore, the energy consumption can be calculated as follows: E = 360 MJ $tonne^{-1}$.

Correspondingly, 46.8 kg tonne^{-1} GHG emitted and 241 kg tonne^{-1} water consumed during the pretreated process.

Processing – REM: The energy consumption for REM is estimated to be 1000 MJ tonne^{-1}, according to the detailed analysis in Supplementary Note 3. Correspondingly, 130 kg tonne^{-1} GHG emitted and 670 kg tonne^{-1} water consumed during the REM process.

Processing – Cyclone separation: Using biochar as the REM conductive additives, the biochar can be recycled by the cyclone separation process, considering the density difference between soil and biochar. During the process, 1 tonne of REM-treated soil mixed with 0.5 tonne of biochar were loaded into the cyclone separator. Generally, a typical cyclone separator can shake 1 tonne of sample per batch and its power is 7.5 kW[16]. Assuming the processing time of 0.25 h, the energy consumption is estimated to be 10.13 MJ tonne^{-1}. Correspondingly, 1.32 kg tonne^{-1} GHG emitted and 6.79 kg tonne^{-1} water consumed during the process.

Processing – Sieving: The sieving separation of metcoke and remediated sample was conducted using an industrial vibrating sieving machine. Generally, a typical shaker machine can shake 0.034 tonne of sample per batch and its power is ~80 W[17]. Assuming the processing time of 0.5 h, the separation energy consumption is estimated to be ~4.2 MJ tonne^{-1}. Correspondingly, 0.55 kg tonne^{-1} GHG emitted, and 2.81 kg tonne^{-1} water consumed during the sieving process.

4.4 Life cycle impact assessment.

In this study, the environmental impacts were classified into three midpoint indicators, including cumulative energy demand (Supplementary Table 13), GHG emission (Supplementary Table 14), and water consumption (Supplementary Table 15).

4.5 Cost evaluation

In this study, the costs for raw materials are from the prices of commercial products, including industrial water ($1.085 per tonne, ref[18]), $KMnO_4$ ($37.7 per kg, ref[19]), KOH ($31.6 per kg, ref[20]), biochar ($1400 per tonne, ref[2]), and metcoke ($150 per tonne, ref[3]). The costs for energy consumption are calculated according to the industrial electricity rate in Texas, US ($0.0587 kWh^{-1}, US Energy Information Administration, ref[1]). The values are listed in Supplementary Table 16. The materials cost and energy cost in electricity are calculated, as shown in Supplementary Table 17. The operating cost is calculated as the sum of the materials cost and energy cost without including labor cost in the operating expense.

4.6 Sensitivity and uncertainty.

There are some uncertainties associated with the energy consumption and GHG emission values of the materials used in this study due to the availability of data from different sources. Although the scalability has been proposed (Supplementary Note 1), REM implemented in this study was conducted on the kilogram scale. There is some uncertainty in the predicted energy consumption for its practical application, especially for the on-site REM process.

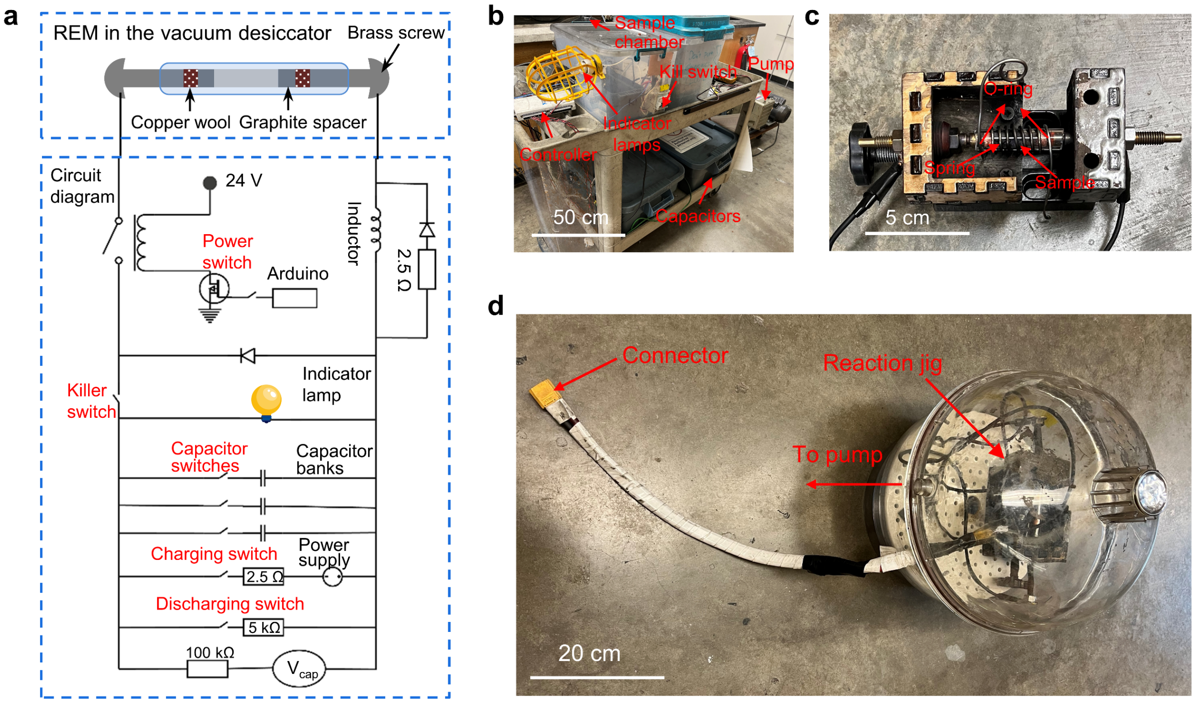

Supplementary Fig. 1. Electrical diagram and pictures of the REM system. a, Electrical diagram of the REM system. The capacitor bank is composed of 10 aluminum capacitors (450 V, 6 mF, Mouser #80-PEH200YX460BQU2) with the total capacitance of 60 mF. More detailed description of the electrical components could refer our previous publications[21,22]. **b,** Picture of the REM setup. **c,** Picture of the REM reaction jig. The graphite electrodes are loaded into the quartz tube, and two brass electrodes with O-rings are used to seal the samples inside the tube. The spring is wound around the tube surface is used to prevent explosion caused by the gas generation during REM process. **d,** Picture of the mild vacuum desiccator chamber.

CAUTION:

To prevent the risk of electrical shock, it is required to follow safety guidelines when operating the equipment. We recommend adhering to the following safety guidelines when using this equipment. For additional safety practices, please refer to our previous publications[22].

1. Enclose or insulate all wire connections securely.
2. Ensure all connections, wires, and components are suitable for high voltages and currents.
3. Be aware that component failure could cause high voltage to appear in unexpected places, such as heat sinks on the switching transistors.
4. Control wires should have opto-isolators rated for high voltage.
5. A visible charge indicator should be provided. A 230 V clear glass incandescent light bulb is a good choice as the glow on the filament also serves as an approximate indicator of the amount of charge on the capacitor bank. Bright light means danger!
6. Avoid using toggle switches with metal toggles. If an arc develops, the metal toggle could become charged and pose a safety risk.
7. Follow the one-hand rule, with one hand working on the system and the other not touching any grounded surface.
8. Provide a mechanical discharge circuit breaker switch connected to a power resistor with a few hundred ohms to rapidly bleed off the capacitor charge.
9. Provide a "kill" circuit breaker switch to disconnect the sample holder from the capacitor bank.
10. Put up high voltage warning signs on the equipment.
11. Remember that the system can discharge thousands of Joules in milliseconds, which can cause components such as relays to explode.
12. Keep a voltmeter with high voltage test leads on hand. When working on the capacitor bank, always check the voltage on each capacitor.
13. Ensure to wear thick rubber gloves extending to the elbows to protect yourself from electrocution when using the equipment.

14. Safety glasses for welding are required to block the infrared and ultraviolet light during the REM reaction.
15. The reliability and robustness of the REM system should be checked by an experienced electrical technician with weekly re-inspections.
16. All users should be properly trained by an experienced electrical technician.

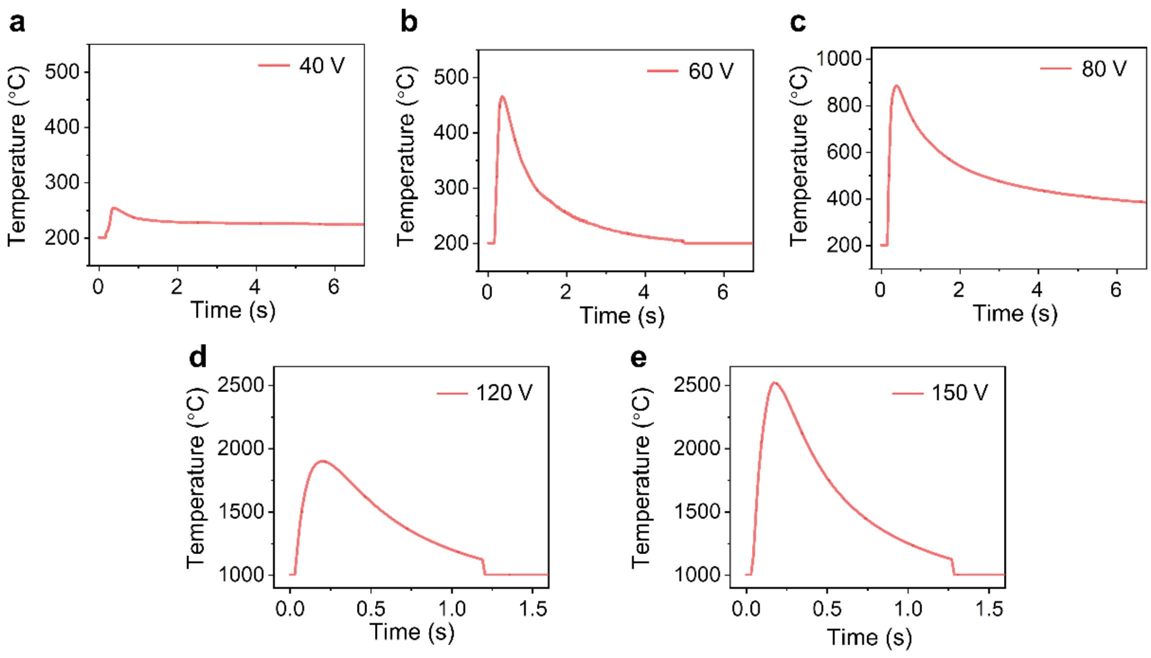

Supplementary Fig. 2. Temperature measurements with different voltage inputs. a, 40 V; **b,** 60 V; **c,** 80 V; **d,** 120 V; and **e,** 150 V.

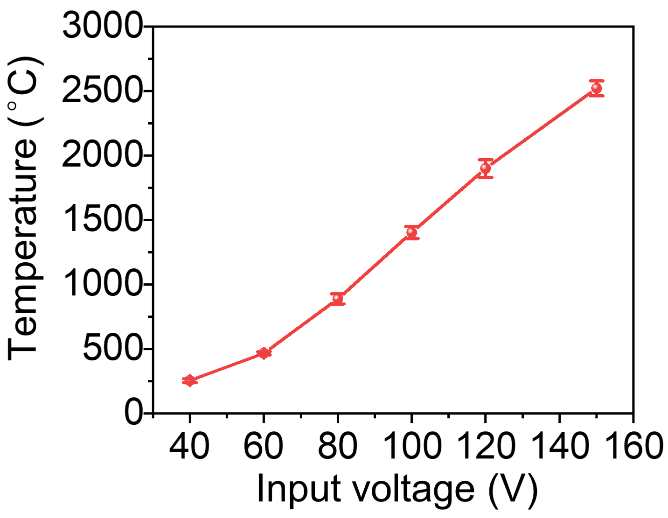

Supplementary Fig. 3. Measured peak temperature varied with input voltage during REM process. The error bars represent the standard deviation, where $N = 3$.

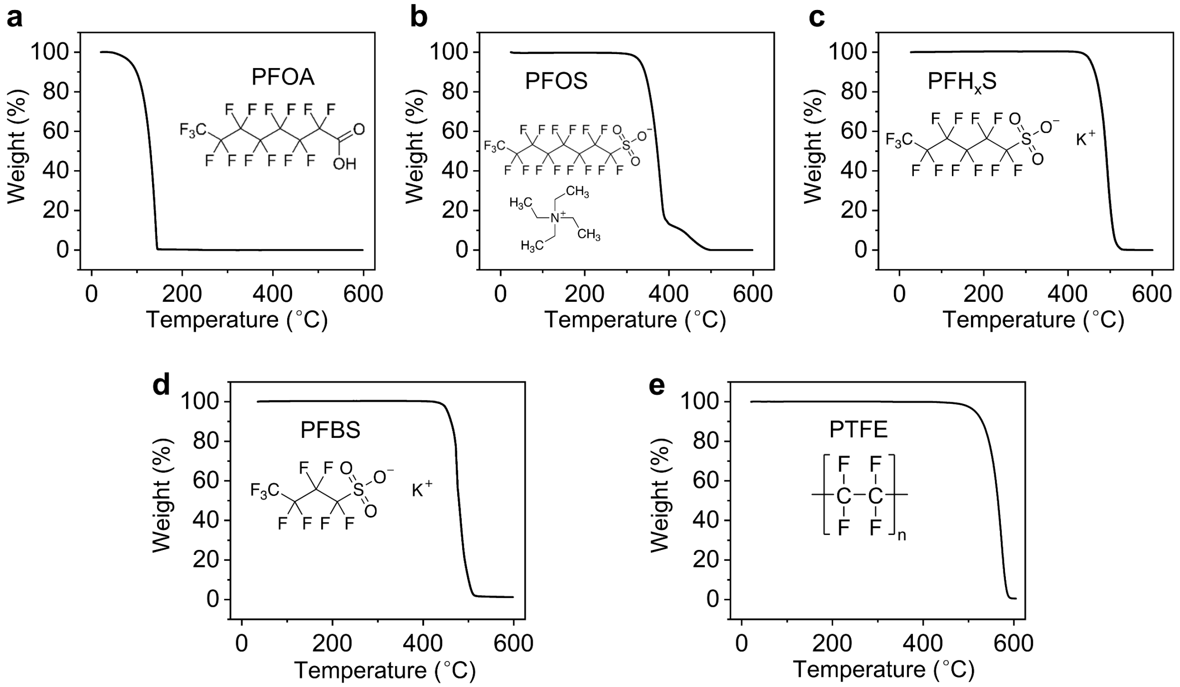

Supplementary Fig. 4. TGA results of PFAS. a, PFOA. **b,** PFOS. **c,** PFH_xS. **d,** PFBS. **e,** PTFE. TGA was conducted in nitrogen with the heating rate of 10 °C min^{-1}. Insets, the chemical structure of each kind of PFAS.

According to the TGA data in Supplementary Fig. 4, the PFAS degradation temperature is usually lower than 600 °C.

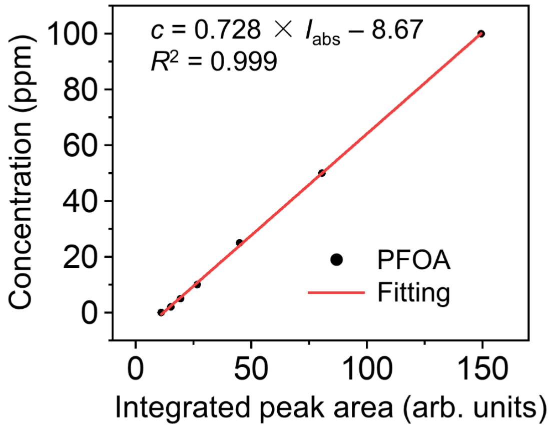

Supplementary Fig. 5. Calibration curve for PFOA by HPLC-DAD. The linearity of the fitting is good ($R^2 > 0.99$), demonstrating the validity of the method for concentration determination.

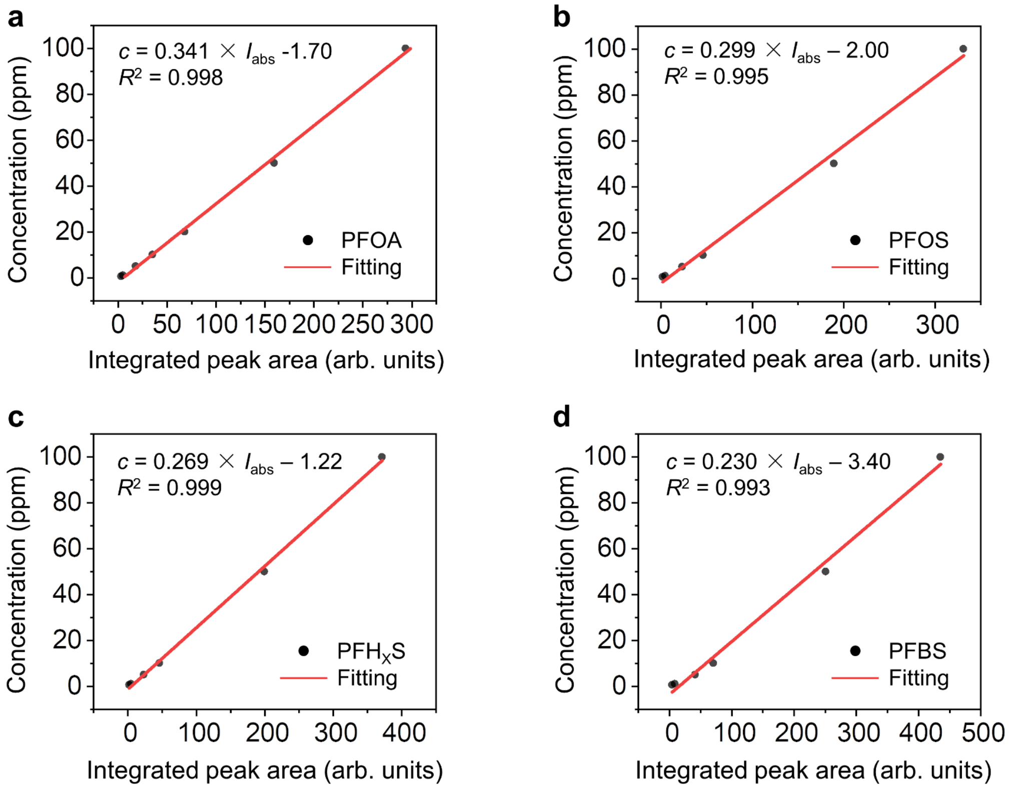

Supplementary Fig. 6. Calibration curves for PFAS by LC-MS. a, PFOA. **b,** PFOS. **c,** PFH_xS. **d,** PFBS. The linearities of all the fittings are good ($R^2 > 0.99$), demonstrating the validity of the methods for PFAS concentration determination.

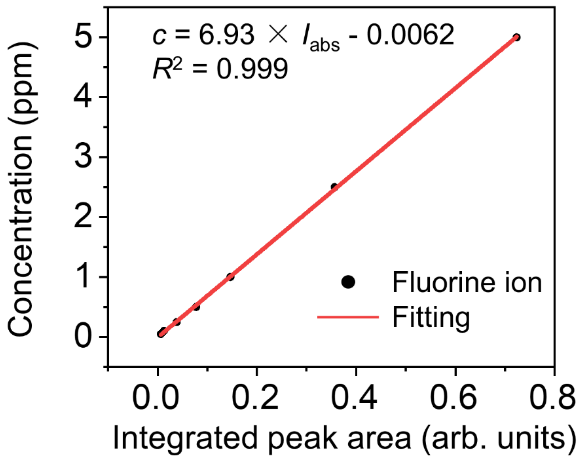

Supplementary Fig. 7. Calibration curve for fluorine ion by IC. The linearity of the fitting is good ($R^2 > 0.99$), demonstrating the validity of the method for concentration determination.

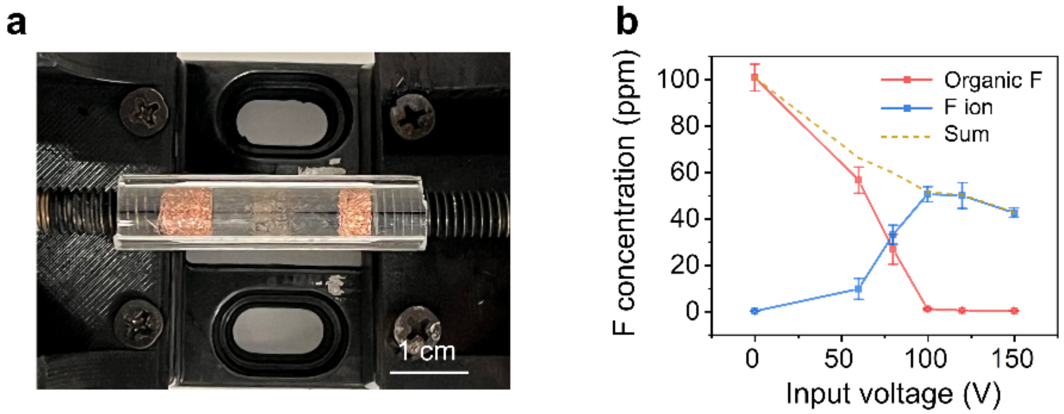

Supplementary Fig. 8. PFOA mineralization in an open system. a, Picture of the REM reaction jig without O-ring sealing. **b**, Concentrations of organic fluorine and mineralized fluorine ion in PFOA-contaminated soil varied with input voltages, conducted in an open REM system without O-rings to seal the quartz tube. The error bars represent the standard deviation, where $N = 3$.

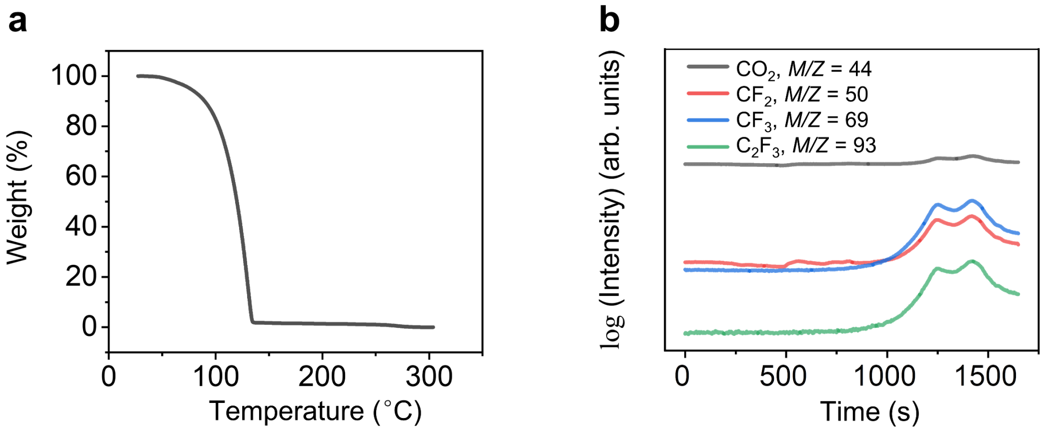

Supplementary Fig. 9. TGA-MS results of PFOA. a, TGA curve. **b,** Corresponding mass spectrum. TGA was conducted in nitrogen with the heating rate of 10 °C min^{-1}. The radicals with mass-to-charge ratio 44, 50, 69, 93 can be assigned to CO_2 (black line), CF_2 (red line), CF_3 (blue line) and C_2F_3 (green line), respectively.

Short-chain fluorocarbon species (C_xF_y) generate and emit to the environment when directly thermally treating PFAS, which are toxic and lead to secondary pollutions.

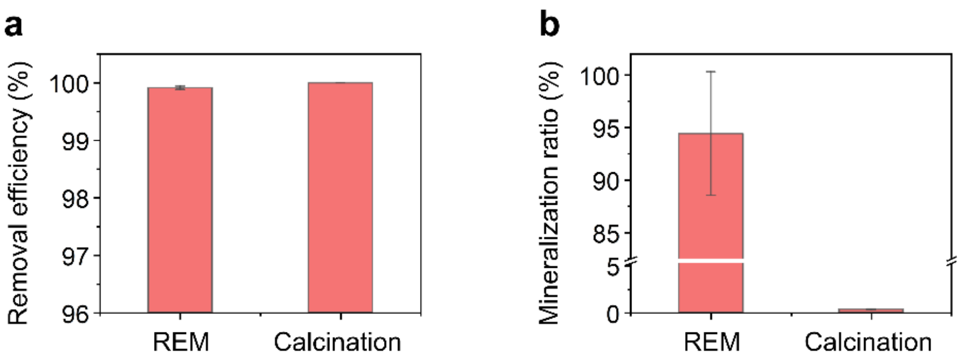

Supplementary Fig. 10. Comparison of PFOA mineralization performance between REM and calcination. a, PFOA removal efficiency. **b,** PFOA mineralization ratio. The error bars in **a** and **b** denote standard deviations, where $N = 3$.

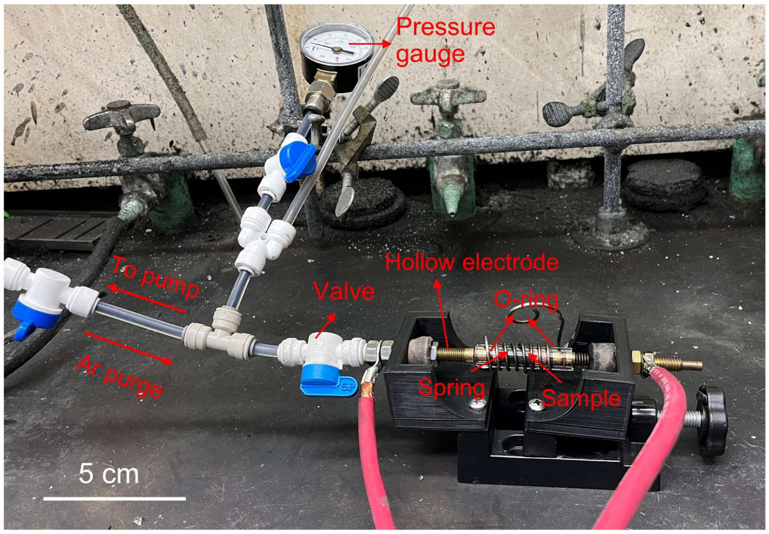

Supplementary Fig. 11. Design for the collection of evolved gas during the REM process.

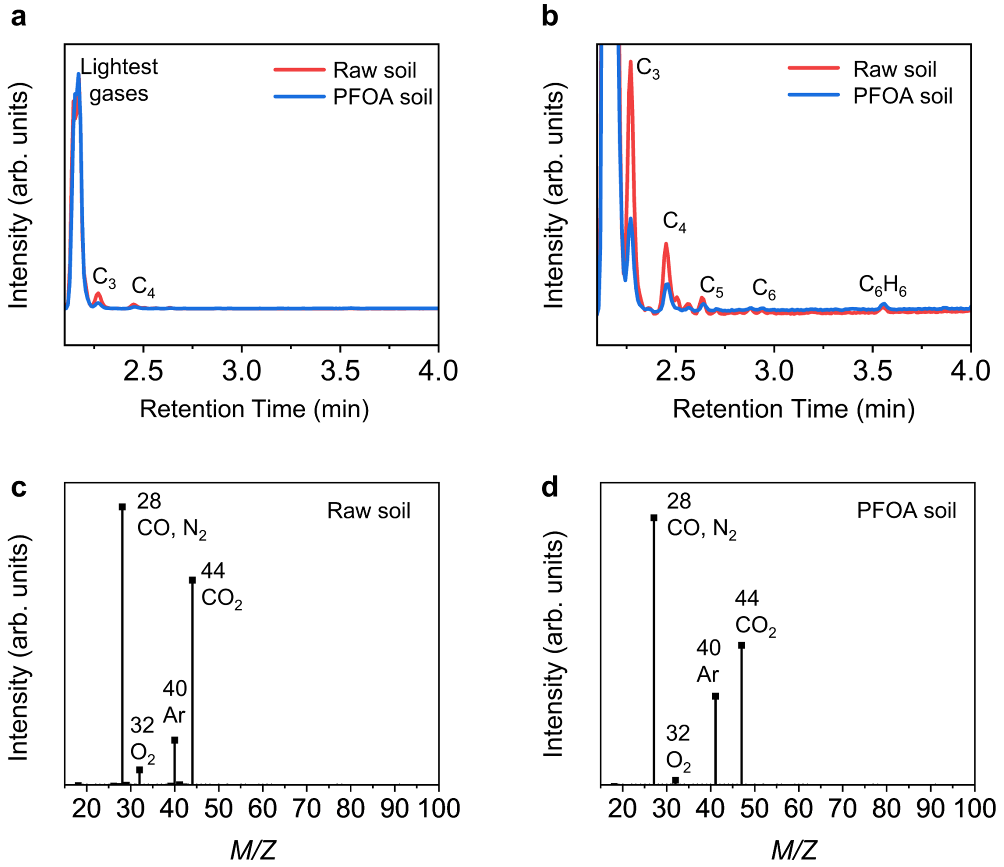

Supplementary Fig. 12. GC-MS results of the soil-evolved gas during the REM process. a, GC-MS chromatograms of the gases for the raw soil (red) and PFOA-contaminated soil (blue). **b,** Zoom-in GC-MS chromatograms. **c,** Mass spectra of the lightest gases for the raw soil. **d,** Mass spectra of the lightest gases for the PFOA-contaminated soil.

In the GC-MS chromatograms, the most intense peak can be ascribed to some lightest gases (Supplementary Fig. 12a,b). Further mass spectra revealed that the main component of the produced volatiles is CO and CO_2, which are produced by the degradation of organic matter in the soil, such as decarboxylation of cellulose or humic acid[23]. The amount of evolved CO_2 exhibits no obvious difference upon the inclusion of PFOA, which can also undergo decarboxylation during REM to evolve CO_2 (Supplementary Fig. 12c,d). Tiny amounts of volatile hydrocarbons such as

propene, 1,3-butadiene, or benzene, are observed for both raw soil and PFOA-contaminated soil, which may also be produced from organic matter in the soil (Supplementary Fig. 9b). No additional peaks corresponding to the mass of PFOA-degraded products or other perfluorinated species, such as HF, F_2, or CF_3, were observed, indicating negligible emission of fluorocarbon species during the sealed REM process.

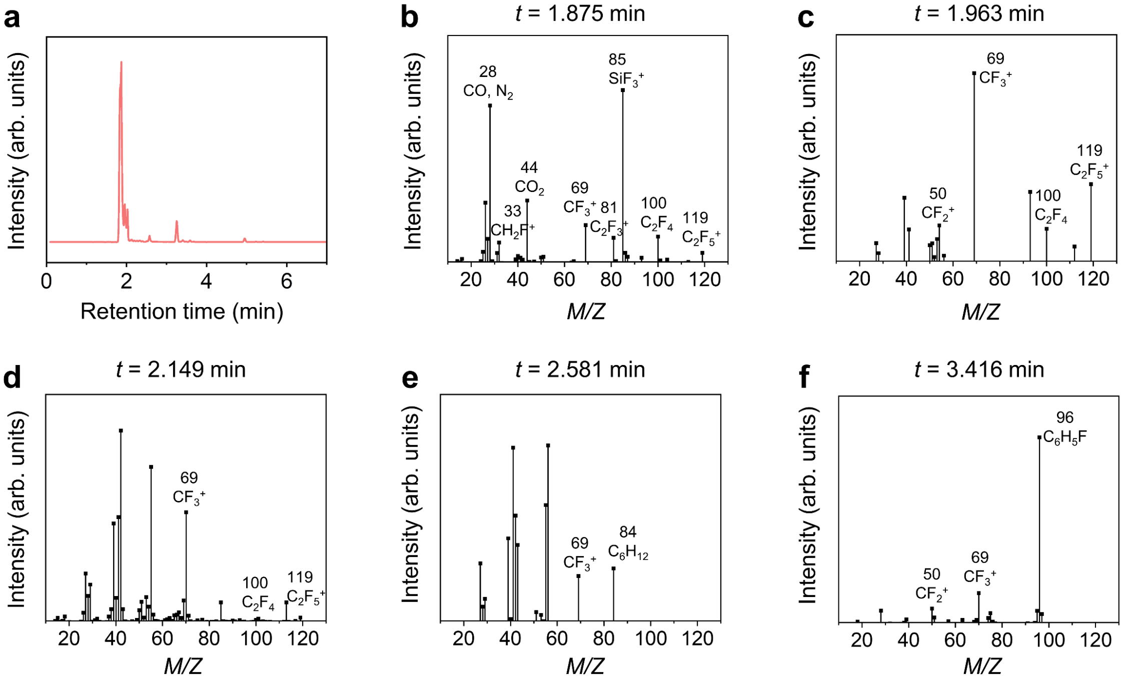

Supplementary Fig. 13. GC-MS results of PFOA-evolved gas without soil during the REM process. a, GC-MS chromatogram of the gases from the mixture of SiO_2 and PFOA. **b-f,** Zoom-in GC-MS chromatograms with different retention times. **b,** 1.875 min; **c,** 1.963 min; **d,** 2.149 min; **e,** 2.581 min; **f,** 3.416 min.

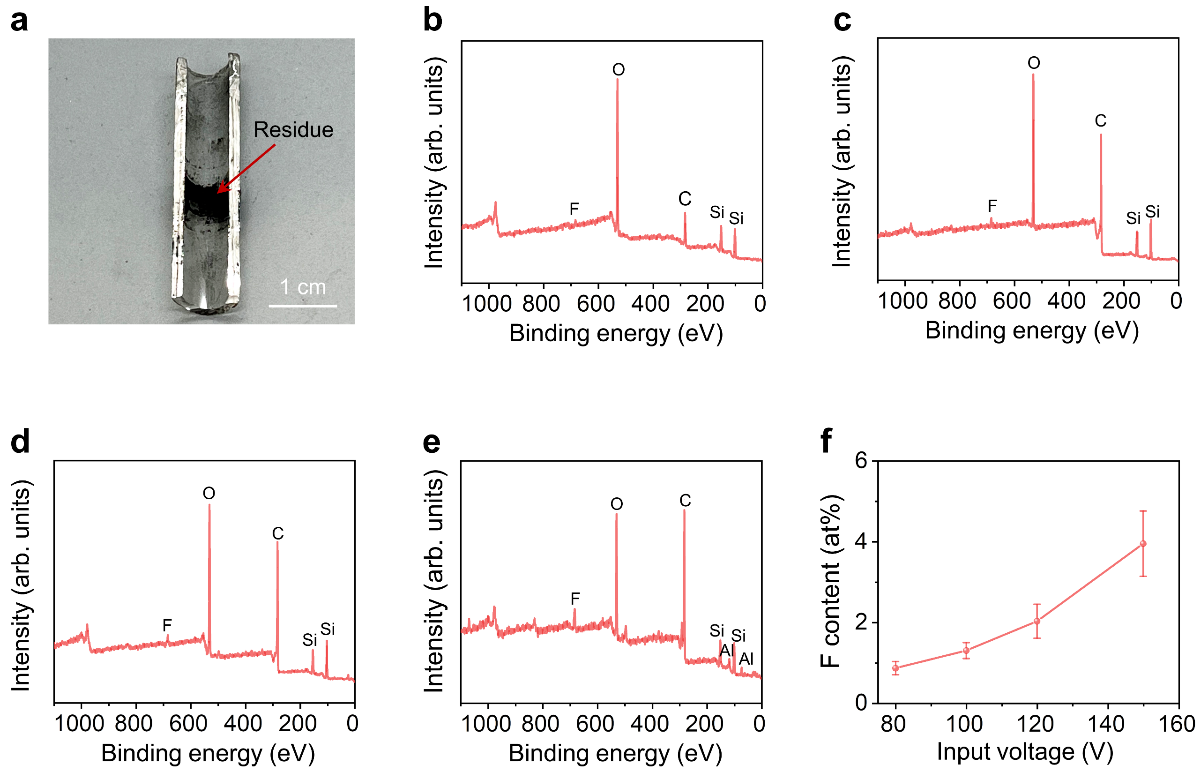

Supplementary Fig. 14. F residue deposited on the inner surface of the quartz tube. a, Picture of the inner surface of quartz tube after REM. **b-e**, XPS spectra of the inner surface of the quartz tube after REM with the input voltage of **b**, 80 V; **c**, 100 V; **d**, 120 V, and **e**, 150 V. **f**, F content on the inner surface of the quartz tube reacted under different input voltages. The error bars in **f** denote standard deviations, where $N = 3$.

Considering the PFOA content in the contaminated soil (~100 ppm) is lower than the XPS detecting limit (~0.1 at%), PFOA with a higher content (~5 wt%) was mixed with soil. After taking out soil samples from the quartz tube reactor, there were some black residues stuck on the inner surface of the quartz tube (Supplementary Fig. 14a), which is hard to dissolve by water. When conducting XPS characterizations on the inner surface of the quartz tube, it was found that its F content increases with the increase of input voltage (Supplementary Fig. 14b-f). It indicates that

more insoluble F-contained compounds were deposited on the quartz tube with higher REM temperature, which influences the complete collection of mineralized F^{-}, and thus decreases the real mineralization ratio, especially under high temperature.

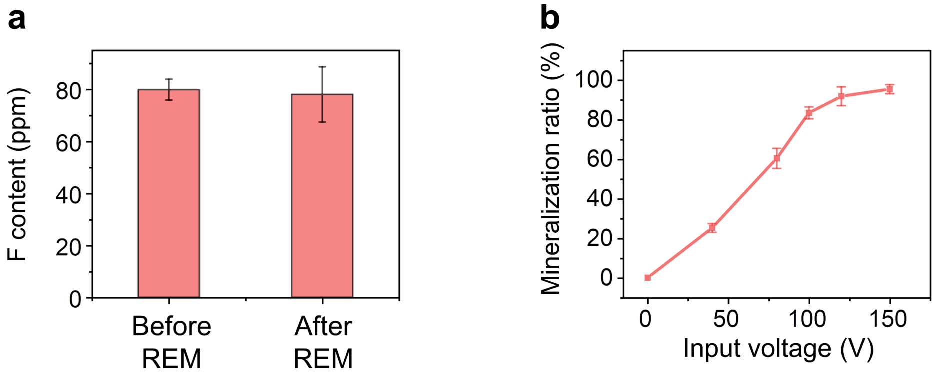

Supplementary Fig. 15. REM for PTFE mineralization. a, Total F content for PTFE-contaminated soil before and after REM. The REM was conducted with an input voltage of 100 V with a duration of 1 s. The total F content was tested by CIC. **b,** PTFE mineralization ratios vary with different input voltage. The error bars in **a** and **b** denote standard deviations, where $N = 3$.

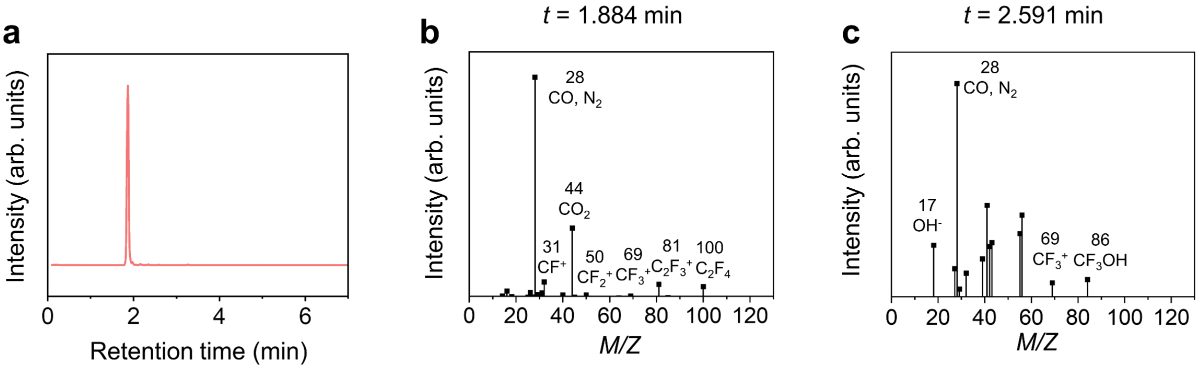

Supplementary Fig. 16. GC-MS results of evolved gas of PTFE-contaminated soil during RE. a, GC-MS chromatogram of the gases from PTFE-contaminated soil. **b-c,** Zoom-in GC-MS

chromatograms with different retention times. **b,** 1.884 min; **c,** 2.591 min. The mass spectra in **b** and **c** correspond to tetrafluoroethylene and trifluoromethanol, respectively.

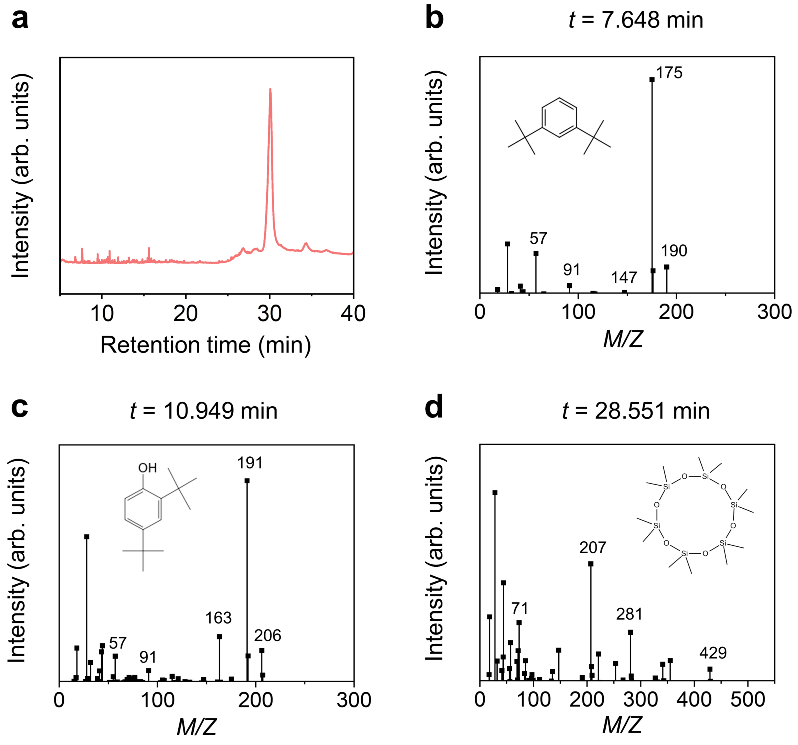

Supplementary Fig. 17. GC-MS results of the solvent-extractant phase of REM soil. a, GC-MS chromatogram of the gases from PTFE-contaminated soil. **b-d,** Zoom-in GC-MS chromatograms with different retention times. **b,** 7.648 min; **c,** 10.949 min; **d,** 28.551 min.

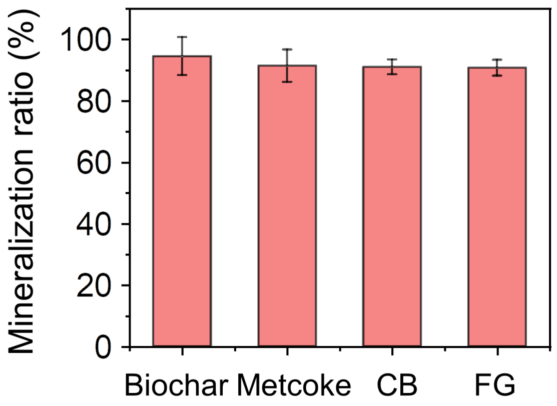

Supplementary Fig. 18. PFOA mineralization ratio with different carbon additives under a REM input voltage of 100 V. The error bars denote standard deviation, where $N = 3$.

When using biochar as the conductive additive, a higher PFOA mineralization ratio can be obtained (~94%), compared with that of other conductive additives (90-91%).

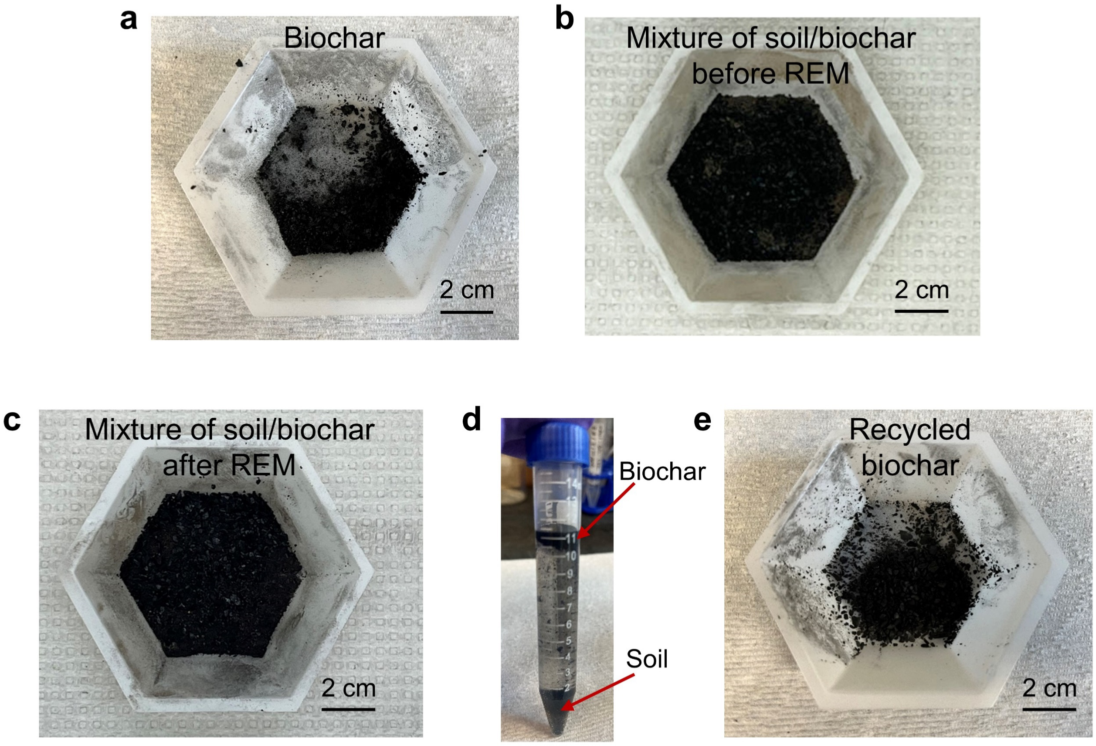

Supplementary Fig. 19. Recycling biochar from REM soil. a, Picture of initial biochar before REM. **b,** Picture of the mixture of soil/biochar before REM process. **c,** Picture of the mixture of soil/biochar after REM process. **d,** REM soil mixed with biochar dispersant after centrifugation, where biochar floated and soil sank. **e,** Picture of recycled biochar after REM, dispersion, centrifugation and drying.

In a typical experiment, we mixed the PFOA-contaminated soil and biochar with a mass of *m*(soil) = 400 mg and *m*(biochar) = 200 mg (Supplementary Fig. 19a,b). After REM process, the remaining mass of mixture was *m*(mixture, REM) = 476 mg (Supplementary Fig. 19c). After centrifugation, filtering and drying (Supplementary Fig. 19d), we obtained purified REM-treated soil with a mass of *m*(purified soil) = 307 mg and recycled biochar with a mass of *m*(recycled biochar) = 169 mg (Supplementary Fig. 19e). Thus, the recycling yield of biochar can be calculated to be ~85%.

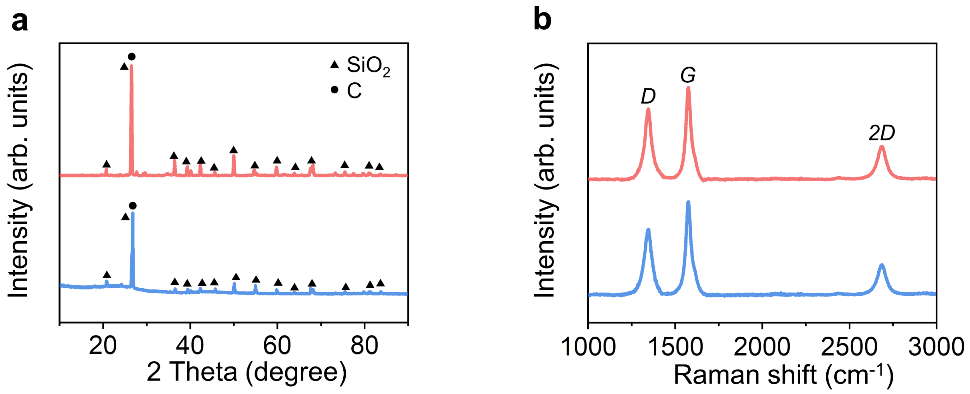

Supplementary Fig. 20. Comparison of biochar and recycled biochar. a, XRD patterns. The PDF reference cards for each are SiO_2, 04-007-0522 (triangle); carbon, 00-056-0159 (circle). **b,** Raman spectra. The red line is the biochar and the blue line is the recycled biochar. No obvious composition differences were found between each other, proving the efficiency of the biochar recycling process.

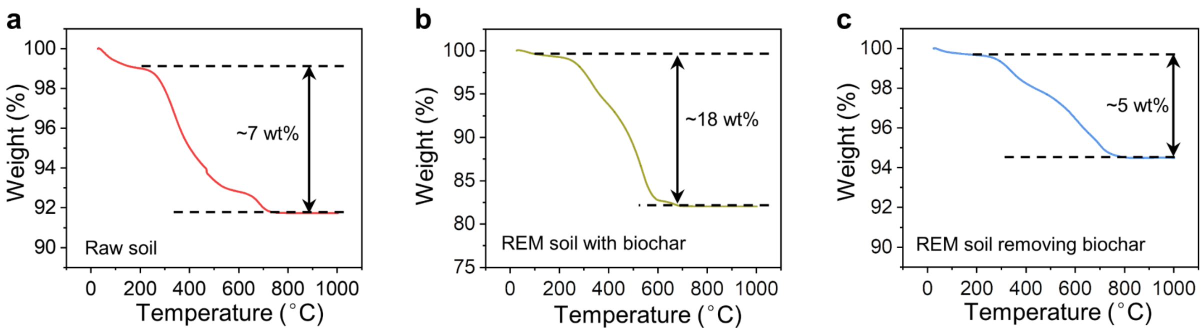

Supplementary Fig. 21. TGA results of soil. a, Raw soil. **b,** REM soil mixed with biochar. **c**, REM soil after removing biochar by centrifugation. TGA test was conducted in air with a heating rate of 10 °C min^{-1}.

For the raw soil, its weight loss comes from the decomposition of organic compounds, which accounts for ~7 wt%. After being mixed with biochar and treated by REM, the soil weight loss increases to ~18 wt%, which results from the oxidation of carbon in biochar. After removing biochar by centrifugation and drying, the weight loss of the REM soil decreased to a low value of ~5 wt%, proving that the majority of the biochar in the soil has been successfully recycled.

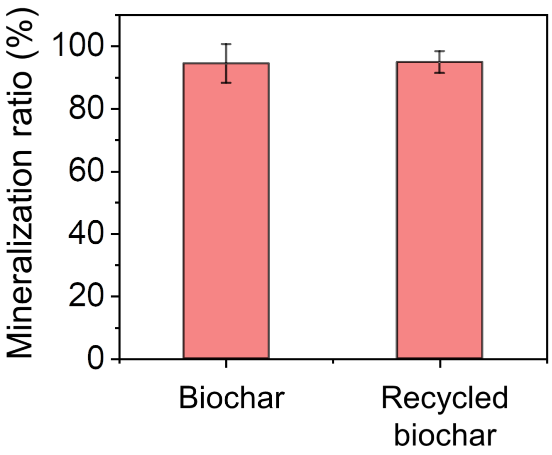

Supplementary Fig. 22. PFOA mineralization ratio using biochar and recycled biochar as the conductive additives after REM treatment. REM process was conducted with an input voltage of 100 V and a duration time of 1 s for one time. The error bars in all plots denote the standard deviation, where $N = 3$.

Negligible change of the PFOA mineralization ratio (~94%) either using biochar and recycled biochar as conductive additives, indicating the efficiency to reuse the recycled biochar in the REM process.

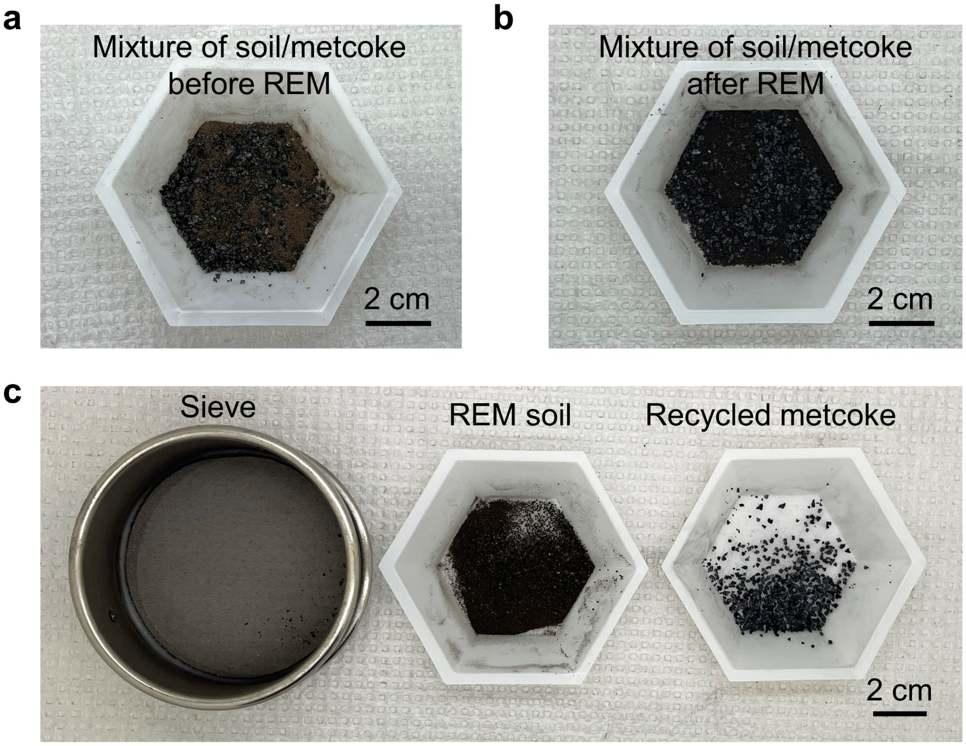

Supplementary Fig. 23. Separation and recycling of the conductive additive, metcoke from soil. a, Picture of the mixture of soil/metcoke before REM process. **b,** Picture of the mixture of soil/metcoke after REM process. **c,** Separation of REM-treated soil and metcoke by sieving.

In a typical experiment, we mixed the PFOA-contaminated soil and metcoke with a mass of *m*(soil) = 400 mg and *m*(metcoke) = 200 mg (Supplementary Fig. 23a). After REM process, the remaining mass of mixture was *m*(mixture, REM) = 473 mg (Supplementary Fig. 23b). After sieving separation, we obtained purified REM-treated soil with a mass of *m*(purified soil) = 287 mg and recycled metcoke with a mass of *m*(recycled metcoke) = 186 mg (Supplementary Fig. 23c). Thus, the recycling yield of metcoke can be calculated to be ~93%.

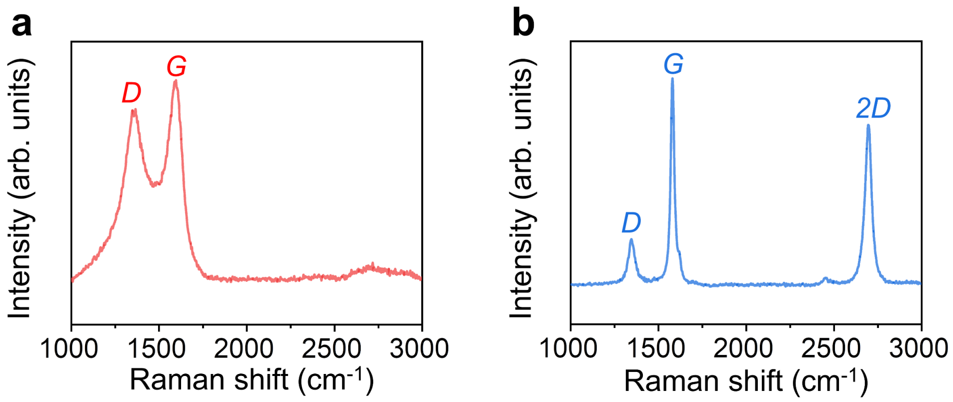

Supplementary Fig. 24. Raman spectra of metcoke and recycled metcoke. a, Initial metcoke before REM. **b,** Recycled metcoke after REM.

Raman spectra showed that the initial metcoke converted to crystalline flash graphene during the REM process.

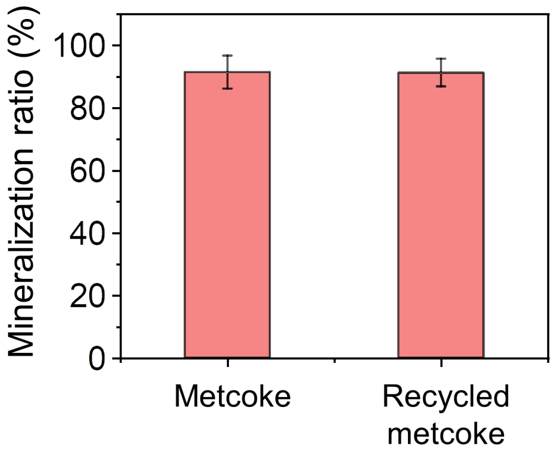

Supplementary Fig. 25. PFOA Mineralization ratio using metcoke and recycled metcoke as the conductive additives after REM treatment. REM process was conducted with an input

voltage of 100 V and the duration time of 1 s for one time. The error bars in all plots denote the standard deviation, where $N = 3$.

When using the recycled metcoke as conductive additives, PFOA mineralization ratio can also reach to a comparable value of ~91%, indicating that the efficiency to reuse the recycled metcoke in the REM process.

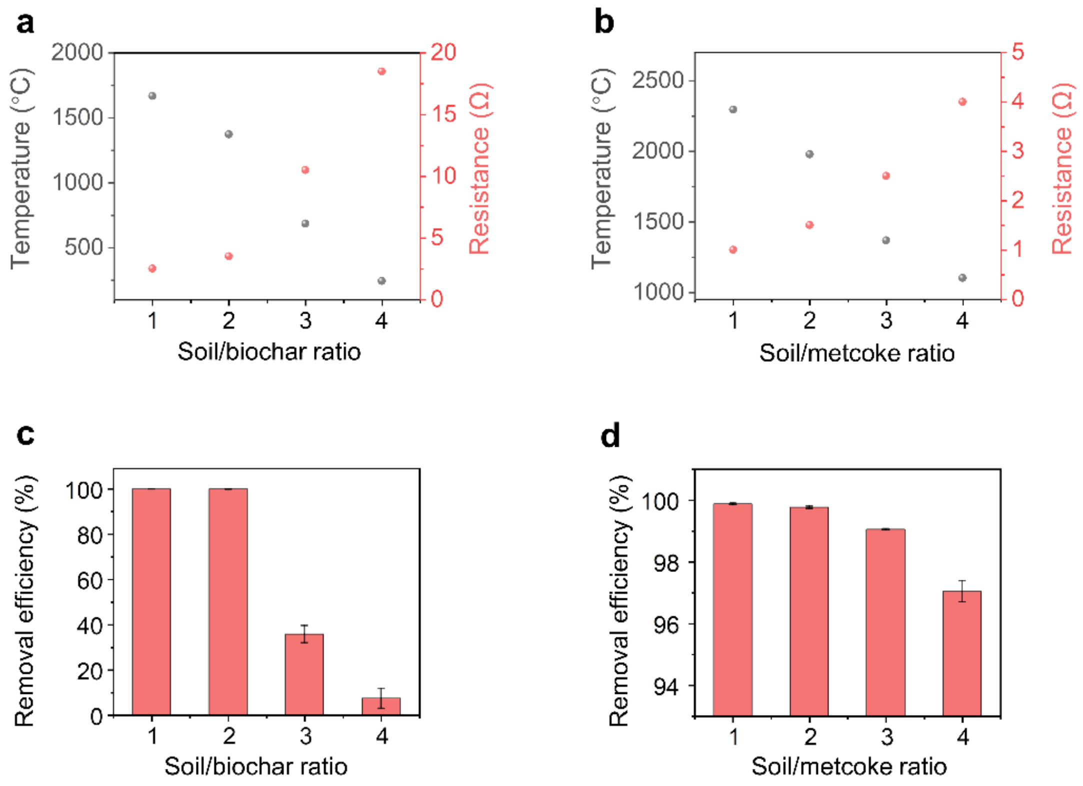

Supplementary Fig. 26. PFOA removal with content of carbon additives by REM. a, Sample resistance and REM temperature with different soil/biochar ratio. **b,** Sample resistance and REM temperature with different soil/metcoke ratio. **c,** PFOA removal efficiency with different soil/biochar ratio. **d,** PFOA removal efficiency with different soil/metcoke ratio. The error bars in **c** and **d** denote standard deviations, where $N = 3$.

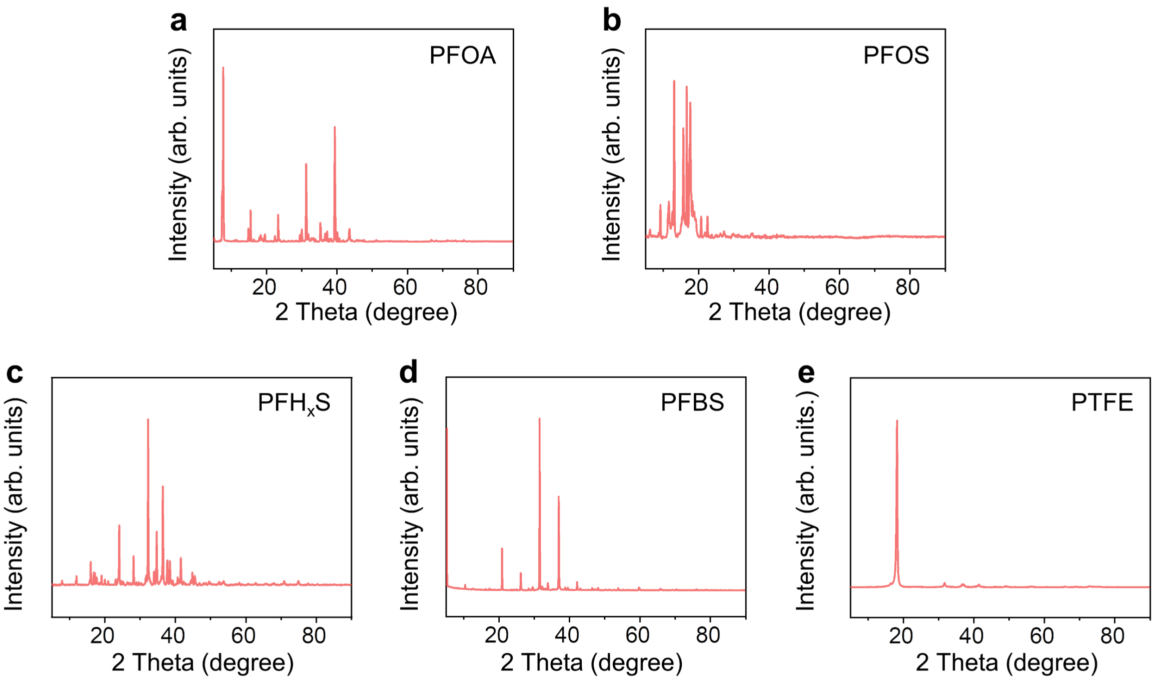

Supplementary Fig. 27. XRD patterns of PFAS. a, PFOA. **b,** PFOS. **c,** PFH$_x$S. **d**, PFBS. **e,** PTFE.

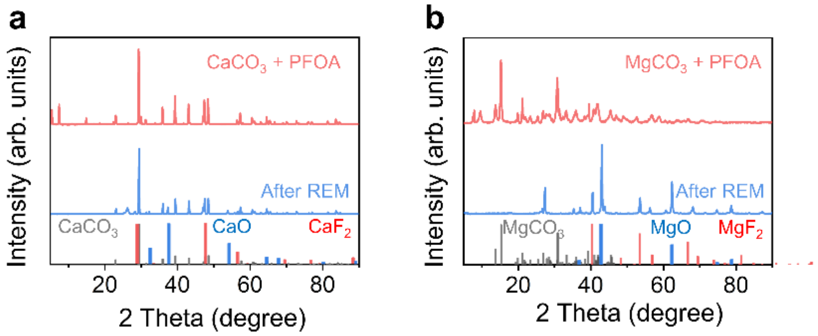

Supplementary Fig. 28. XRD patterns of different metal carbonates mixed with PFAS before (red line) and after (blue line) REM. a, $CaCO_3$. **b,** $MgCO_3$. **c,** Na_2CO_3. The PDF reference cards for each are $CaCO_3$, 01-085-0849; CaO, 00-048-1467; CaF_2, 04-008-4867; $MgCO_3$, 00-025-0513; MgO, 01-076-2583; MgF_2, 00-041-1443; Na_2CO_3, 00-037-0451; NaF, 01-080-8614.

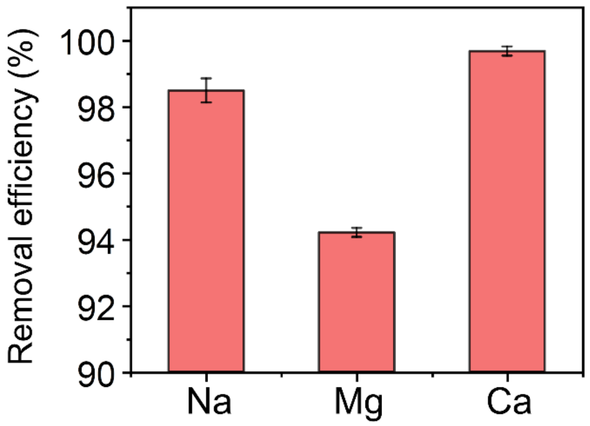

Supplementary Fig. 29. PFOA removal efficiency by mixing different metal carbonates with PFOA for REM. The error bars denote standard deviations, where $N = 3$.

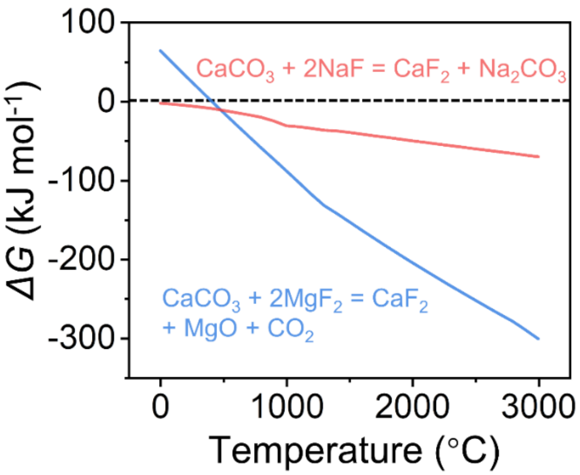

Supplementary Fig. 30. The Gibbs free energy change (ΔG) of the conversion from other metal fluorides (red line for NaF and blue line for MgF_2) to CaF_2 at different temperatures.

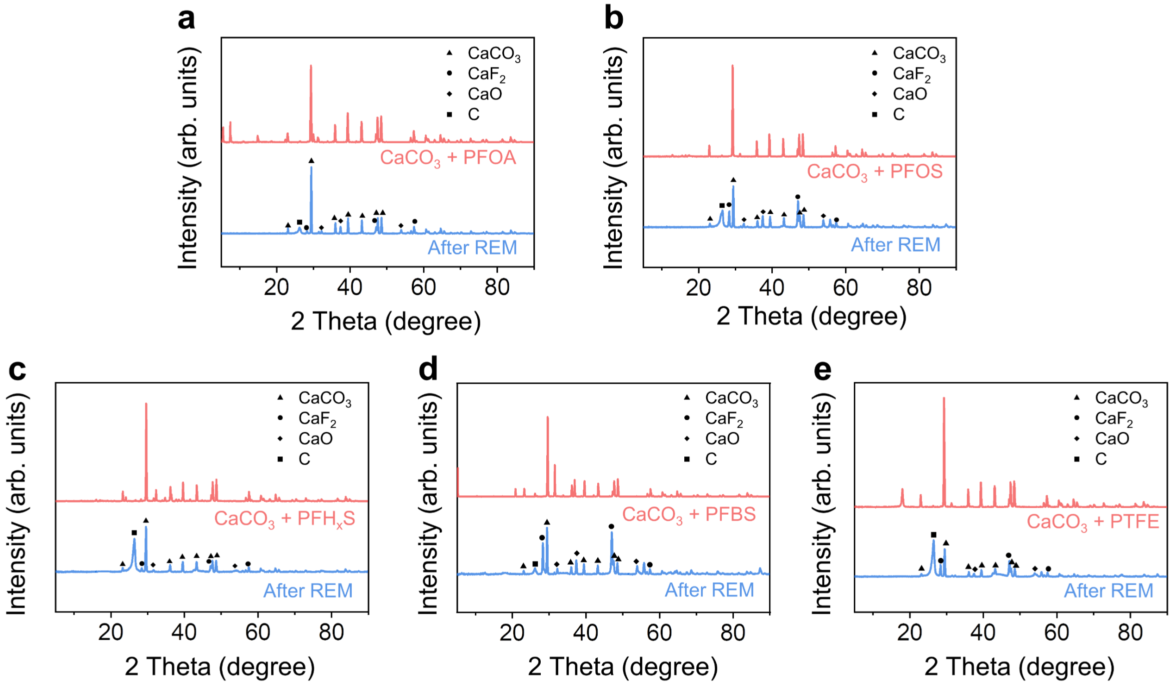

Supplementary Fig. 31. XRD patterns of the $CaCO_3$ mixed with PFAS before (red line) and after (blue line) REM. a, PFOA-mixed $CaCO_3$. **b**, PFOS-mixed $CaCO_3$. **c**, PFH_xS-mixed $CaCO_3$. **d**, PFBS-mixed $CaCO_3$. **e**, PTFE-mixed $CaCO_3$. The PDF reference cards for each are $CaCO_3$, 01-085-0849; CaF_2, 04-008-4867; CaO, 00-048-1467; and carbon, 00-056-0159.

Considering that $CaCO_3$ is the main calcium specie in soil, we directly mixed the $CaCO_3$ with different types of PFAS (PFOA, PFOS, PFH_xS, PFBS, and PTFE) with a Ca/F mole ratio of 0.6 (see details from Supplementary Table 3) to ensure 20 at% excessive supply of Ca, where metcoke (with undetectable Ca) was used as carbon additives during REM process.

For each kind of PFAS, their original XRD peaks vanished (Supplementary Figs. 27 and 31) and the CaF_2 peak appeared after the REM treatment (Supplementary Fig. 31). The CaO peak may originate from the decomposition of $CaCO_3$. These data evinced that the existence of Ca in the soil facilitates the PFAS mineralization during REM process.

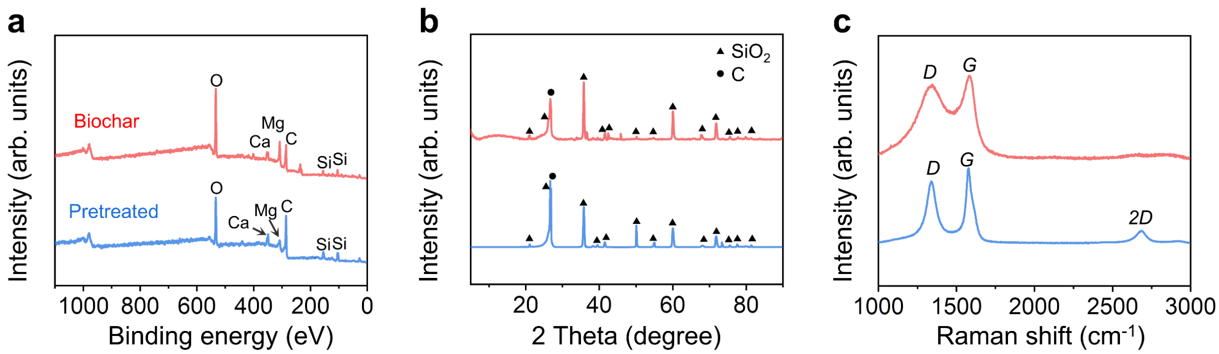

Supplementary Fig. 32. Characterization of biochar. a, XPS full spectra of raw biochar (red) and pretreated biochar (blue). **b**, XRD patterns of raw biochar (red) and pretreated biochar (blue). The PDF reference cards for each are SiO_2, 04-007-0522 (triangle); carbon, 00-056-0159 (circle). **c**, Raman spectra of raw biochar (red) and pretreated biochar (blue).

After the pretreatment, the elemental components and crystal structures of the biochar show negligible changes (Supplementary Fig. 32a,b), where SiO_2 and carbon are the major crystal components of the biochar. The Ca content in biochar is ~4 at% measured by XPS (Supplementary Fig. 32a). However, the crystallinity of the carbon improved significantly, indicated by the narrow XRD peak at ~26° (Supplementary Fig. 32b) and the appearance of 2*D* peak in the Raman spectrum (Supplementary Fig. 32c), which benefits the enhancement of its conductivity and makes it more suitable to be used as the conductive additives for further REM process. Note that the following term "biochar" refers to the pretreated biochar if without specific notification.

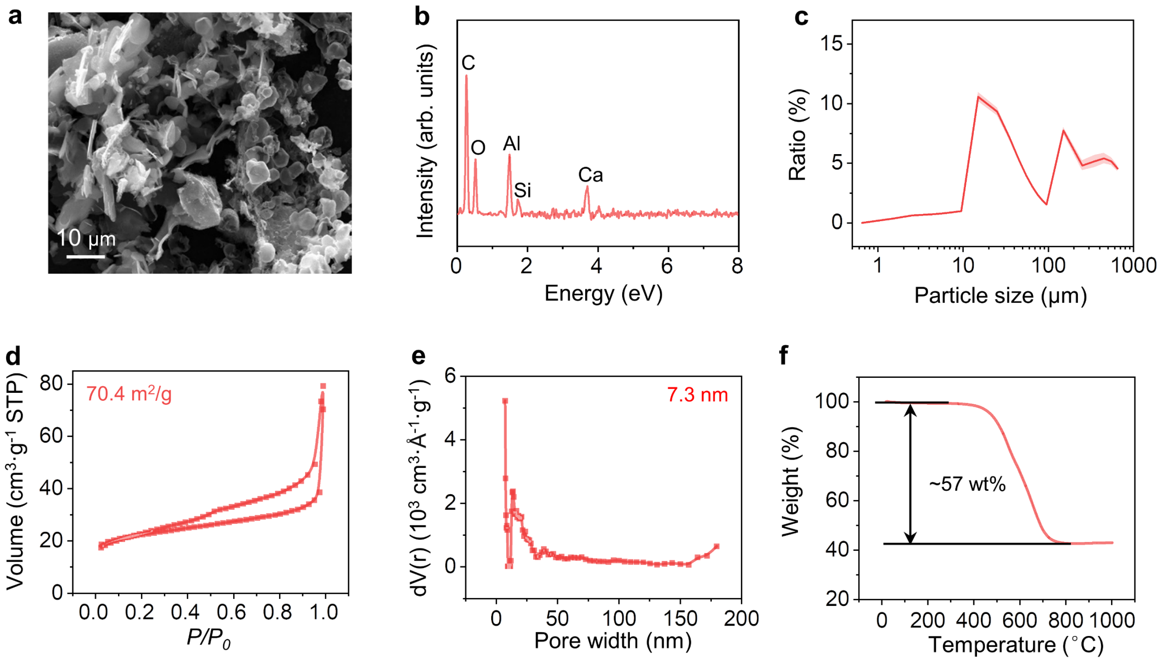

Supplementary Fig. 33. Characterizations of pretreated biochar. a, SEM image. **b**, EDS spectrum. **c**, Particle size distribution. The shadow denotes the standard deviation, where $N = 5$. **d**, N_2 adsorption-desorption isotherms. **e,** Pore width distribution calculated by density functional theory (DFT) model. **f,** TGA curve. TGA was conducted in air with the heating rate of 10 °C min^{-1}.

The biochar exhibits a porous sheet structure with a higher content of silicon, aluminum, calcium than conventional carbon additives (Supplementary Fig. 33a,b), which consists well with its XPS data (Supplementary Fig. 32a). The biochar has a wide size distribution from ~10 to ~1000 μm (Supplementary Fig. 33c). According to the Brunauer–Emmett–Teller (BET) result, it has a large specific area (70.4 m^2 g^{-1}) and small pore width (7.3 nm, Supplementary Fig. 33d,e). The TGA result shows ~57 wt% weight loss of biochar under the air (Supplementary Fig. 33f). Considering the biochar mainly consists of carbon and quartz (Supplementary Fig. 32b), the TGA weight loss can be ascribed to oxidation of carbon in the air, which indicates that biochar contains ~57 wt% carbon (Supplementary Fig. 33f).

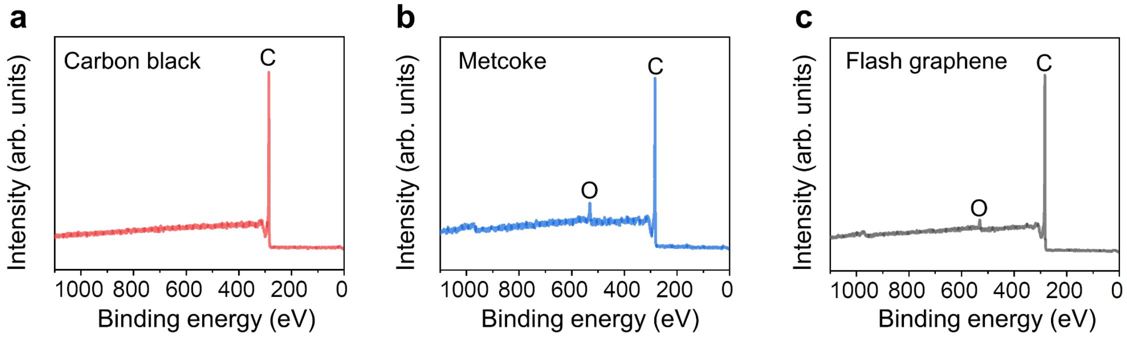

Supplementary Fig. 34. XPS full spectra of different conductive additives. a, Carbon black. **b,** Metcoke. **c,** Flash graphene (FG) derived from metcoke. Except carbon and oxygen, no other elements were detected in their XPS spectra.

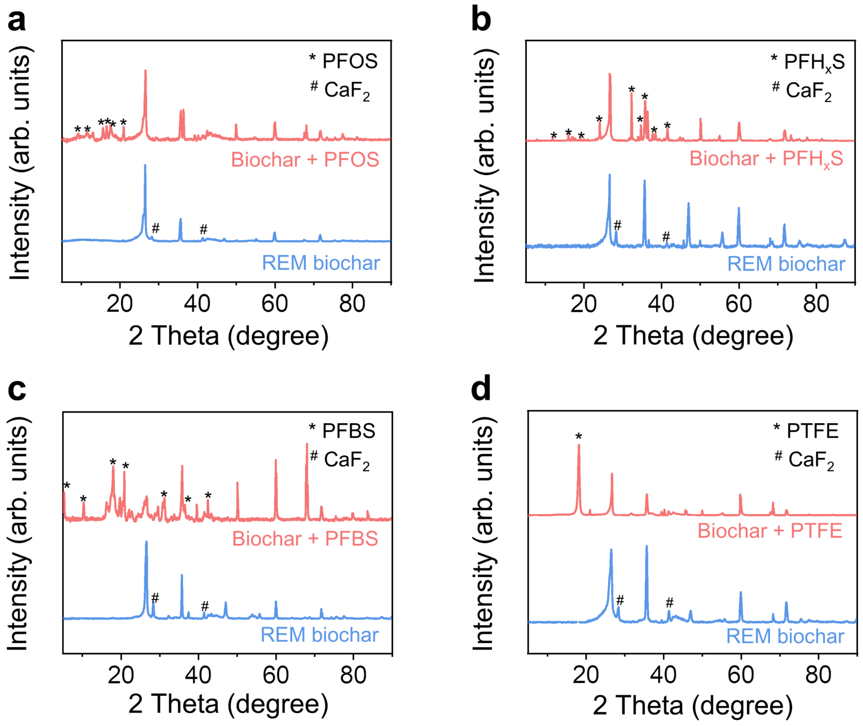

Supplementary Fig. 35. XRD patterns of the biochar mixed with PFAS before (red) and after (blue) the REM process. a, PFOS-mixed biochar. **b,** PFH_xS-mixed biochar. **c,** PFBS-mixed biochar. **d,** PTFE-mixed biochar. The PDF reference cards for CaF_2 is 04-008-4867.

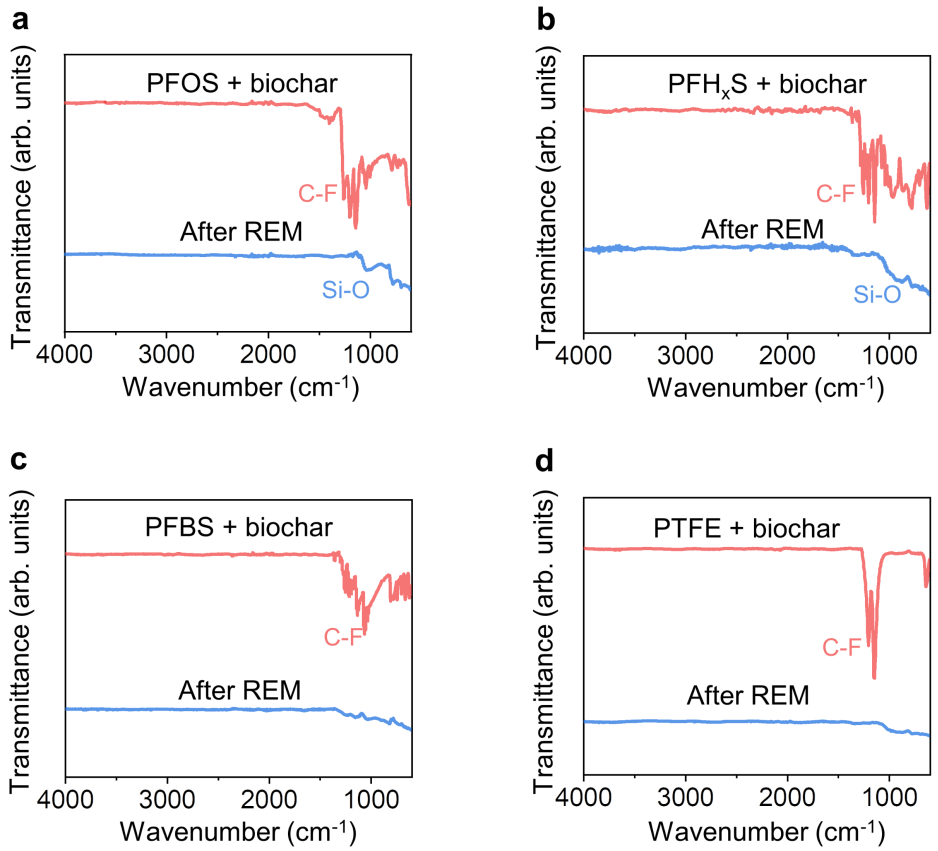

Supplementary Fig. 36. IR spectra of the biochar mixed with PFAS before (red) and after (blue) the REM process. a, PFOS-mixed biochar. **b,** PFH_xS-mixed biochar. **c,** PFBS-mixed biochar. **d,** PTFE-mixed biochar.

Like the PFOA-mixed biochar in Fig. 3a, the XRD spectra reveal that original peaks of all kinds of PFAS vanish while the CaF_2 peak appear after the REM process (Supplementary Fig. 35). The distinct C-F stretching vibration peaks (1100-1200 cm^{-1}) of PFAS in IR spectra[24] also disappear after REM (Fig. 3d and Supplementary Fig. 36). These indicate that the Ca-species can react with organic fluorine to form CaF_2 during the REM process.

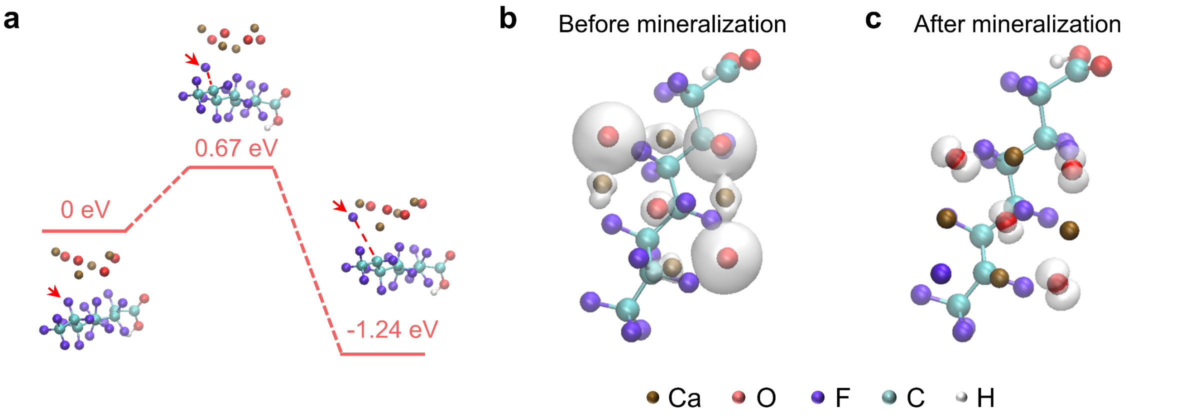

Supplementary Fig. 37. Simulation results of PFOA mineralization with the presence of Ca. **a**, Kinetic barrier of an F atom removal from a PFOA molecule with the help of Ca. Insets are the structures of the reactant, transition state, and product, and the red arrow marked the F atom being removed from the PFOA and then joining the CaO cluster during the process. **b**, Initial structure and partial charge density of the CaO and PFOA molecules. **c**, Final structure and partial charge density with the removal of one F atom from the PFOA.

The highly diffusive charge density of the highest occupied molecular orbital (HOMO) states (around the Fermi level at -2.6 eV) is localized in the CaO, indicating its high chemical reactivity before mineralization. In comparison, the Fermi level of the final structure decreases to -4.8 eV, and the electron states in the F^- are lower than the HOMO states. This indicates with the presence of Ca, F is more favorable to ionically bond with Ca atoms than forming a covalent bond with the C atom.

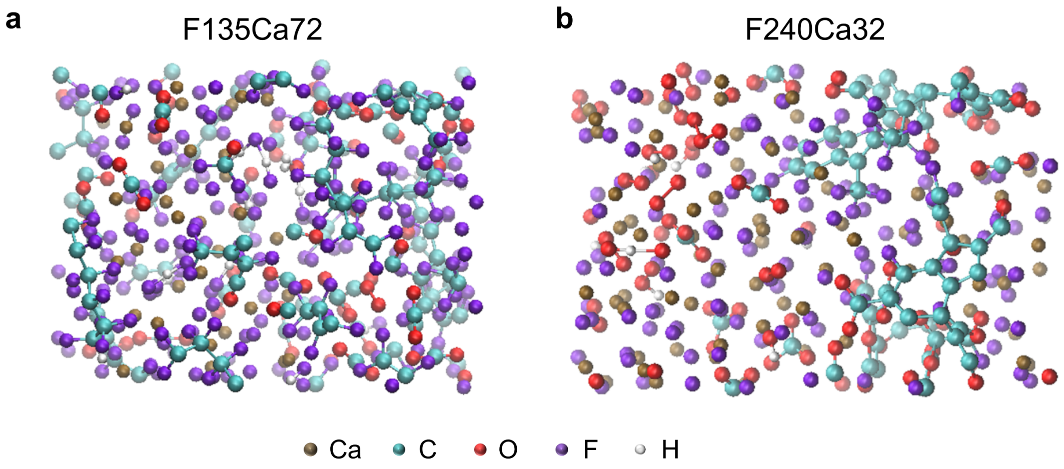

Supplementary Fig. 38. Optimized structure snapshots of mixed calcium oxide and PFOA with different Ca/F mole ratio under the thermal treatment in the temperature range of 1500 K to 2500 K. a, F240Ca32. **b,** F135Ca72.

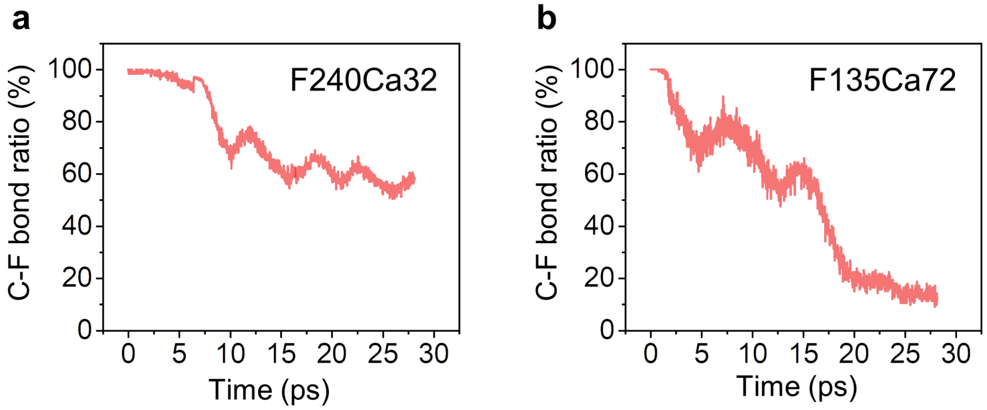

Supplementary Fig. 39. MD simulated C-F bond ratio variation with different Ca/F mole ratio with the thermal treatment at ~2000 K. a, F240Ca32. **b,** F135Ca72.

According to the simulation results (Fig. 3f-h, Supplementary Figs. 38 and 39), C-F ratio significantly decreases with the increase of Ca/F ratio, indicating that the existence of Ca^{2+} can

facilitate the cleavage of C-F bonds, and thus reduce the emission of harmful PFOA-degraded short-chain fluorocarbon compounds.

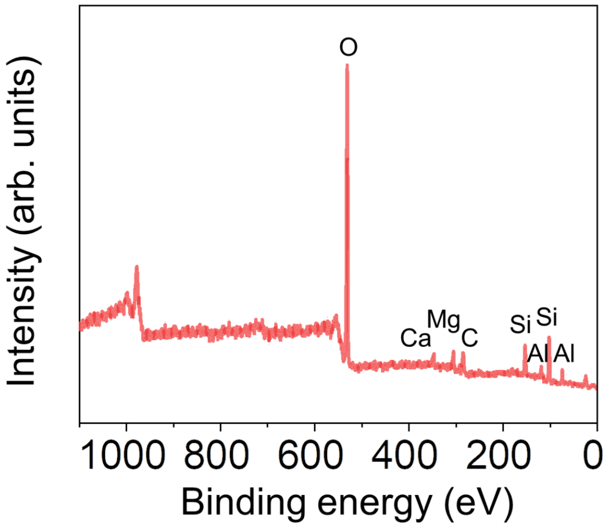

Supplementary Fig. 40. XPS full spectrum of raw soil.

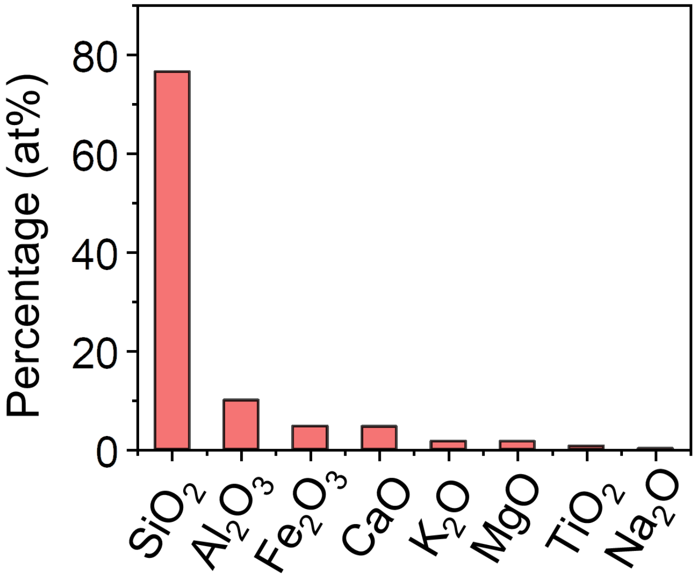

Supplementary Fig. 41. XRF result of raw soil.

The XPS spectrum and XRF result reveal that raw soil contains a Ca content (4-5 at%, Supplementary Figs. 40 and 41), while biochar also has a comparable Ca content of ~4% (Supplementary Fig. 32a). Considering the fluorine mass content in PFAS-contaminated soil is ~100 ppm, the required mass content of Ca was calculated to be ~105 ppm to mineralize PFAS into CaF_2 according to the stoichiometric ratio. Therefore, Ca contents in both soil and biochar are much excessive, more than two orders of magnitude higher than the required content for PFAS mineralization.

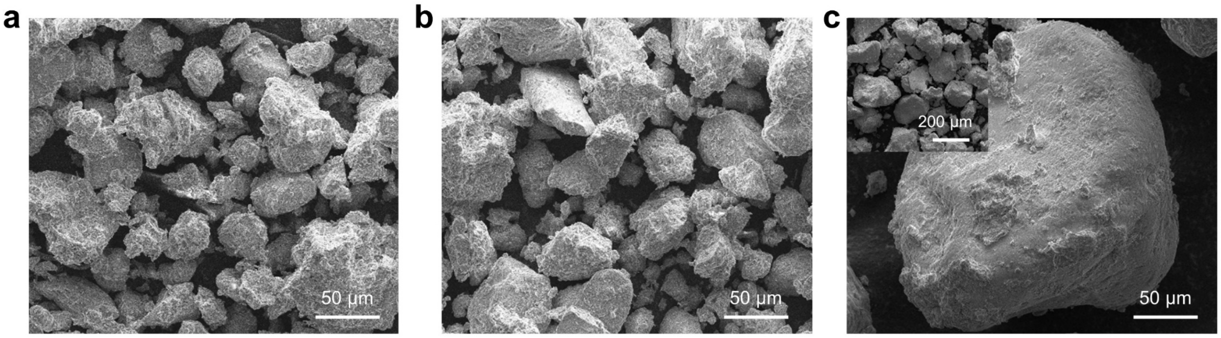

Supplementary Fig. 42. Soil morphology characterization. a, SEM image of raw soil. **b,** SEM image of REM soil. **c,** SEM image of calcined soil. Inset, low-magnified SEM image of calcined soil particles.

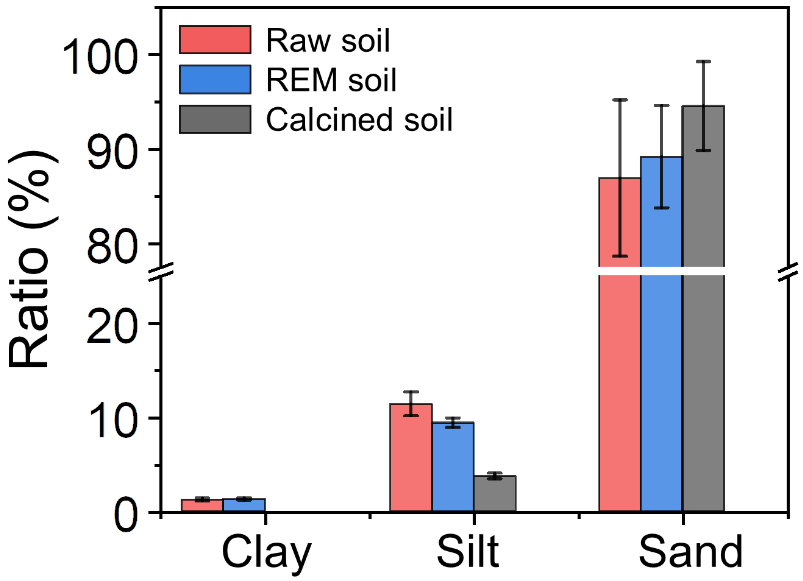

Supplementary Fig. 43. The ratio of soil particle types for raw soil (red), REM soil (blue), and calcined soil (black) based on particle size analysis data. Herein, the particles with size <2 μm are catalogued as clay, while the size in the range of 2-50 μm for silt, and the size >50 μm for sand. The error bar denotes standard deviation, where $N = 5$.

After REM process, the soil particle size and morphology exhibit negligible changes compared with those of raw soil (Supplementary Fig. 42a,b). In contrast, after calcination, the soil particles show dozens-time enlargement (Supplementary Fig. 42c). Further laser particle analysis proved that the maintenance of particle sizes after REM and the increase of particle sizes after calcination (Fig. 4b). Moreover, based on the statistical results in Supplementary Fig. 43, the raw soil and REM soil have similar clay, silt and sand ratio. However, the clay ratio for the calcined soil decreases to zero, while its silt ratio decreases by 67%.

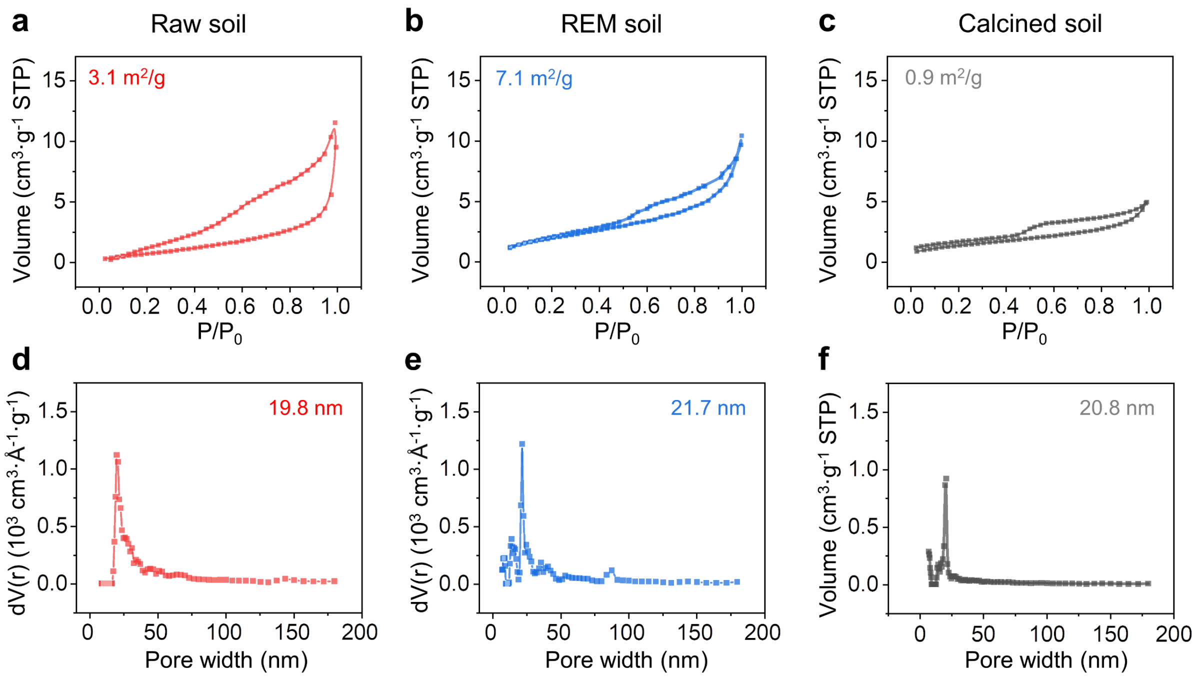

Supplementary Fig. 44. BET measurement of soil. a-c, N_2 adsorption-desorption isotherms at 77 K. **a,** Raw soil, **b,** REM soil, and **c,** calcined soil. **d-f,** Pore width distribution calculated by DFT model. **d,** Raw soil, **e,** REM soil, and **f,** calcined soil. Note that the surface area and pore width of REM soil are measured after removing the biochar by centrifugation.

Different from the calcined soil with a significantly decreased surface area, REM soil particles exhibit a comparable and even larger surface area than raw soil particles, proving that REM did not cause the aggregation and enlargement of soil particles. The increased surface area of REM soil may come from the residual biochar, which has a higher specific area than soil (Supplementary Fig. 33d,e).

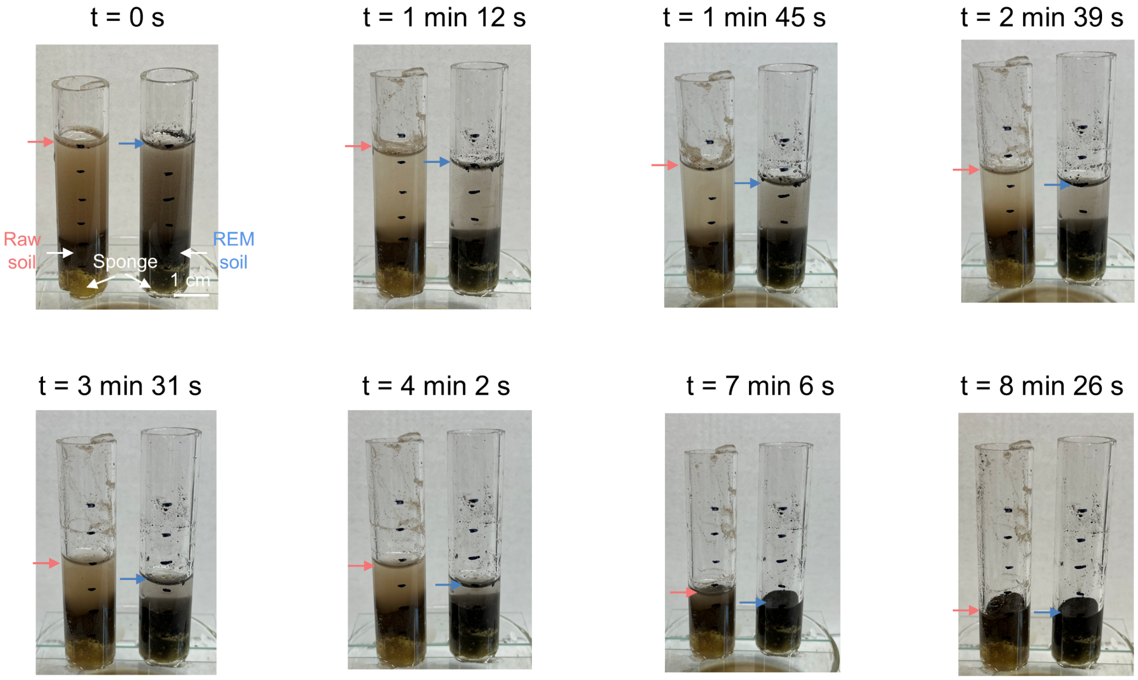

Supplementary Fig. 45. Water infiltration test of raw soil and REM soil. Pictures of water levels with time. The red arrow and blue arrow show the liquid levels of raw soil and REM soil, respectively.

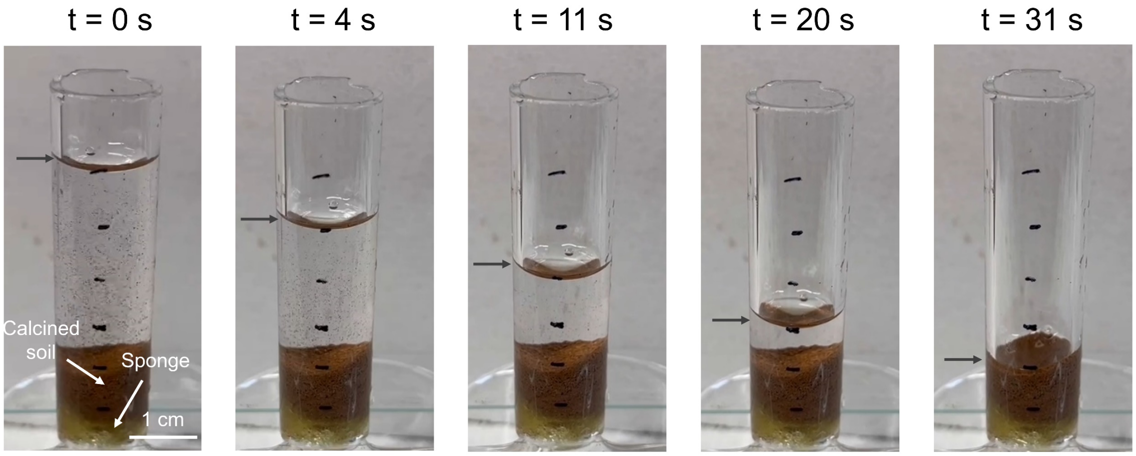

Supplementary Fig. 46. Water infiltration test of the calcined soil. Pictures of water levels with time. The black arrow shows the liquid levels of calcined soil.

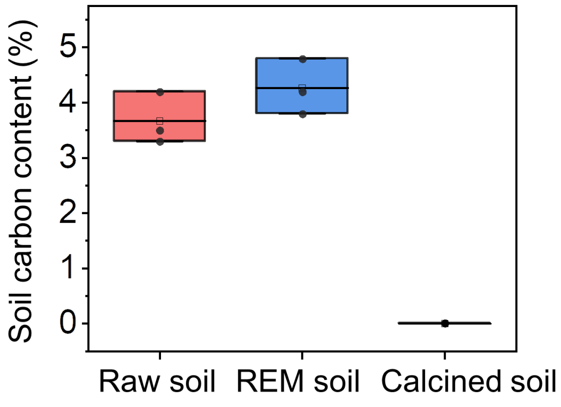

Supplementary Fig. 47. Soil carbon content in the raw soil, REM soil and the calcined soil. The central line in the boxplot represents the median value and the box limits represent the upper and lower quartiles. The whiskers represent 5th and 95th percentiles. 3 parallel experiments were conducted for each sample. REM soil has a comparable soil carbon content with raw soil, while the soil carbon content in the calcined soil is lower than the detecting limit (<0.1 wt%).

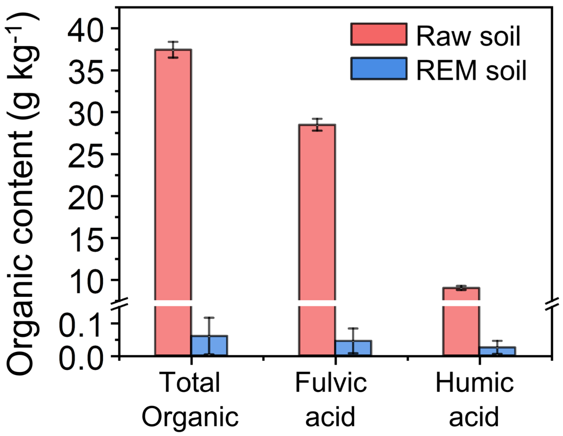

Supplementary Fig. 48. Organic content in raw soil (red) and REM soil (blue). The error bars denote standard deviations, where $N = 3$

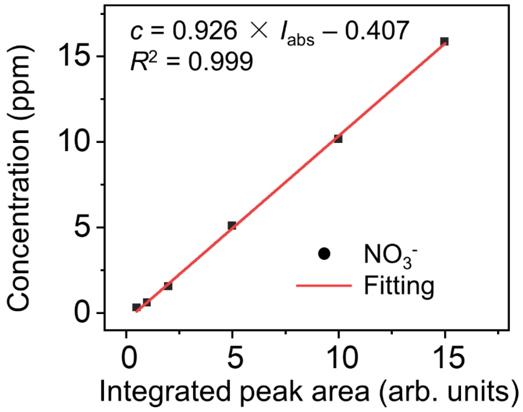

Supplementary Fig. 49. Calibration curve for nitrate concentration by IC. The linearity of the fitting is good (R^2 > 0.99), demonstrating the validity of the method for concentration determination.

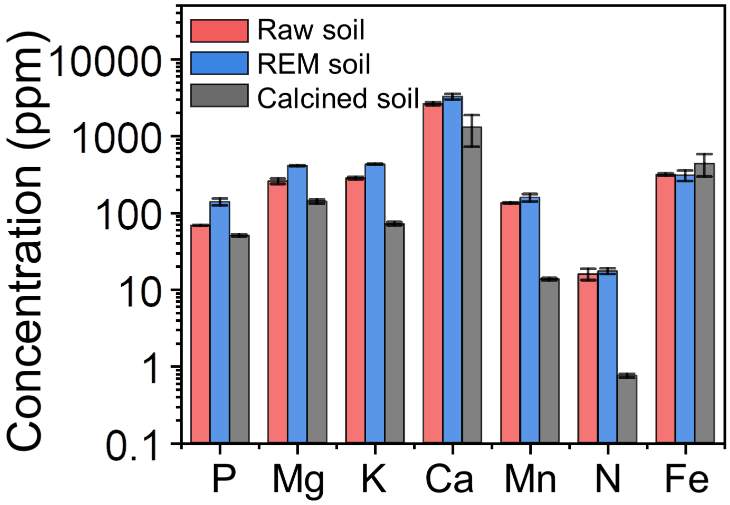

Supplementary Fig. 50. Soil nutrients concentration measurement. The exchangeable contents of Ca, Fe, P, N, Mn, K, and Mg in the raw soil (red), REM soil (blue) and calcined soil (black). The error bars represent the standard deviations, where $N = 3$.

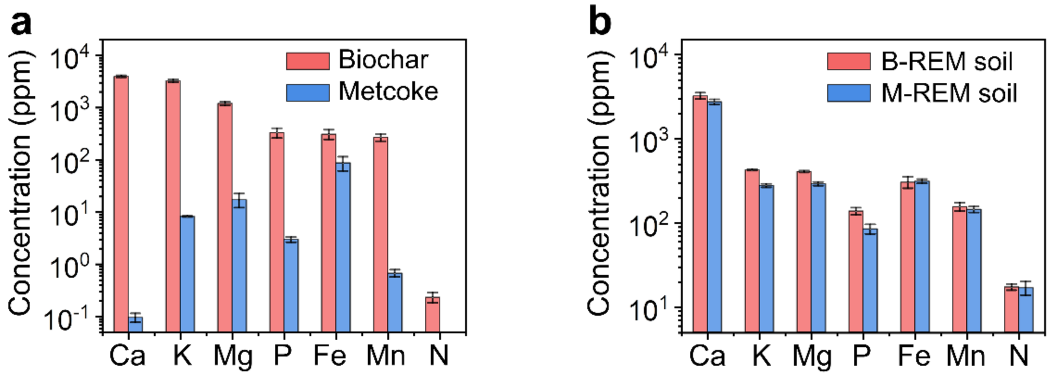

Supplementary Fig. 51. Soil nutrient concentration measurements using different carbon additives. a, Exchangeable nutrient contents of Ca, Fe, P, N, Mn, K, and Mg in the biochar (red) and metcoke (blue). **b,** Exchangeable nutrient contents of REM soil using biochar (B-REM soil, red) and metcoke (M-REM soil, blue) as the carbon additives. The error bars represent the standard deviations, where $N = 3$.

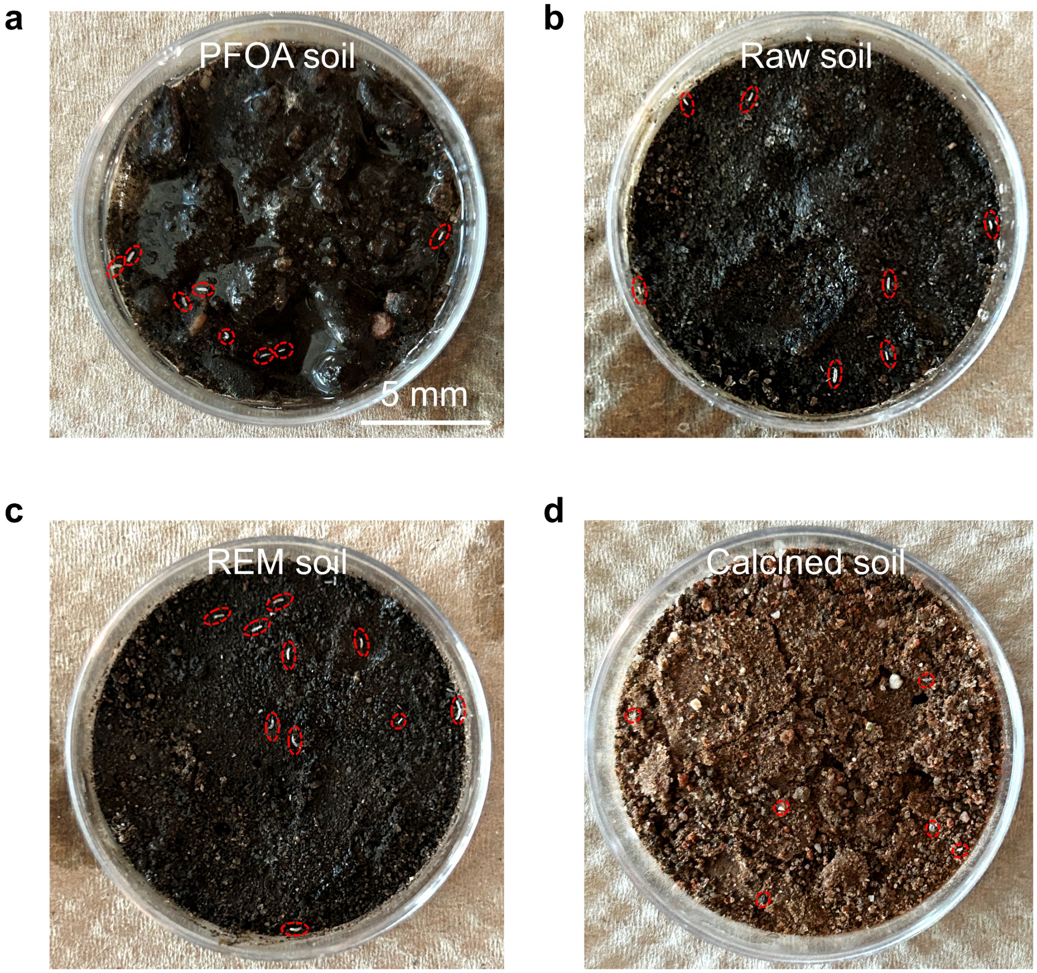

Supplementary Fig. 52. Pictures of Petri dishes with different soil samples for springtail culture. a, PFOA soil. **b,** Raw soil. **c,** REM soil. **d,** Calcined soil. The red dash circle denotes the springtails in each Petri dish.

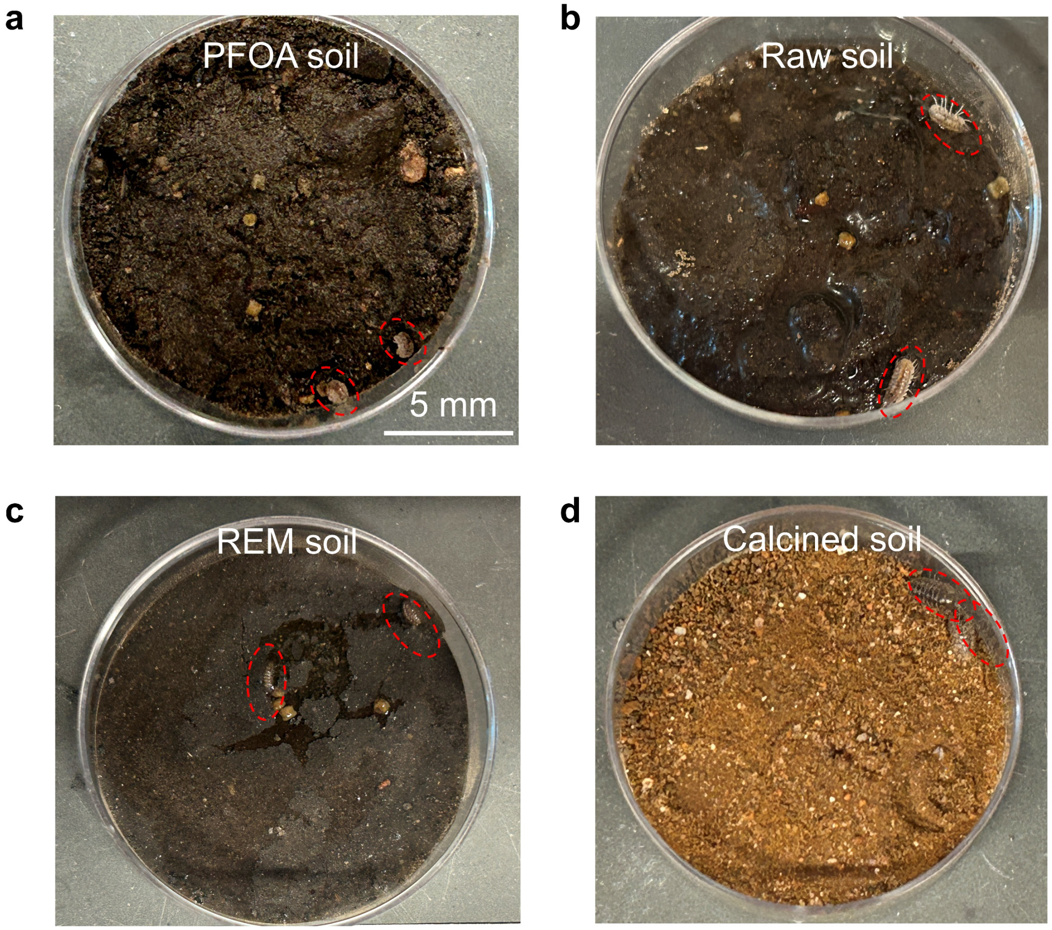

Supplementary Fig. 53. Pictures of Petri dishes with different soil samples for isopod culture. **a,** PFOA soil. **b,** Raw soil. **c,** REM soil. **d,** Calcined soil. The red dash circle denotes the isopods in each Petri dish.

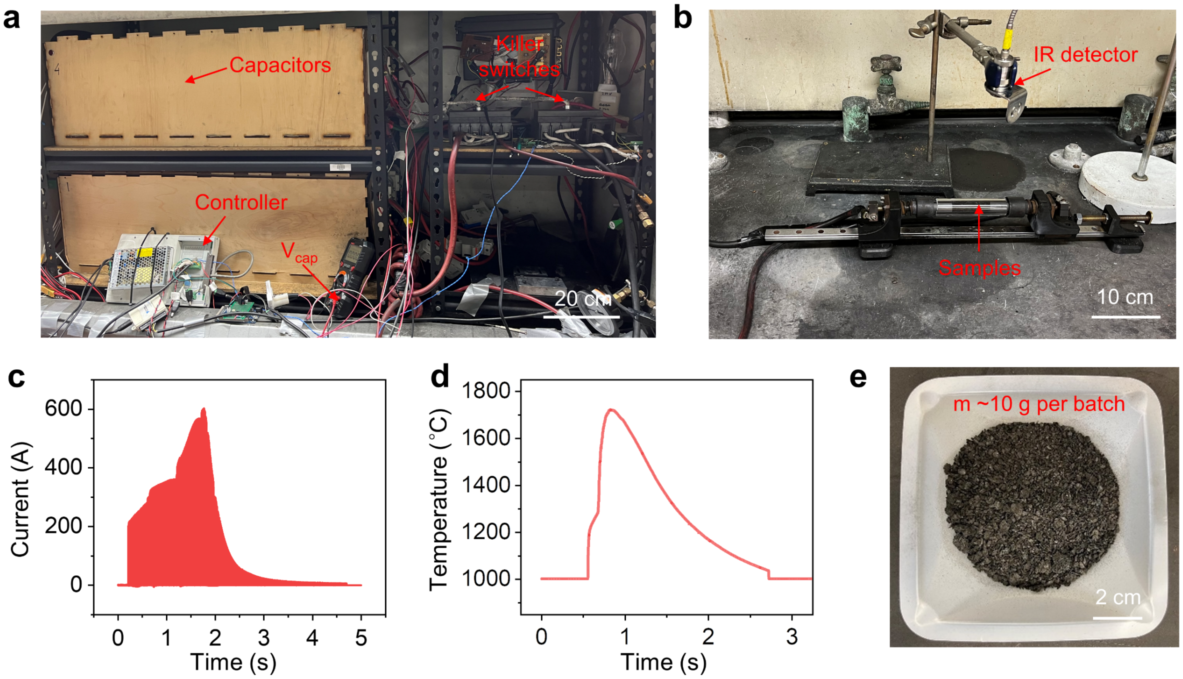

Supplementary Fig. 54. Scaling up of the REM process for gram-scale soil remediation. a, Picture of the gram-scale REM equipment with C_1 = 0.624 F. **b,** Picture of the reaction stage for large-scale samples. **c,** Current profile under input voltage of 300 V. **d,** Real-time temperature curve of the large-scale sample recorded by an infrared thermometer. The temperature detection range of the thermometer is 1000 - 3000 °C. **e,** REM soil sample mixed with biochar of 10 g per batch.

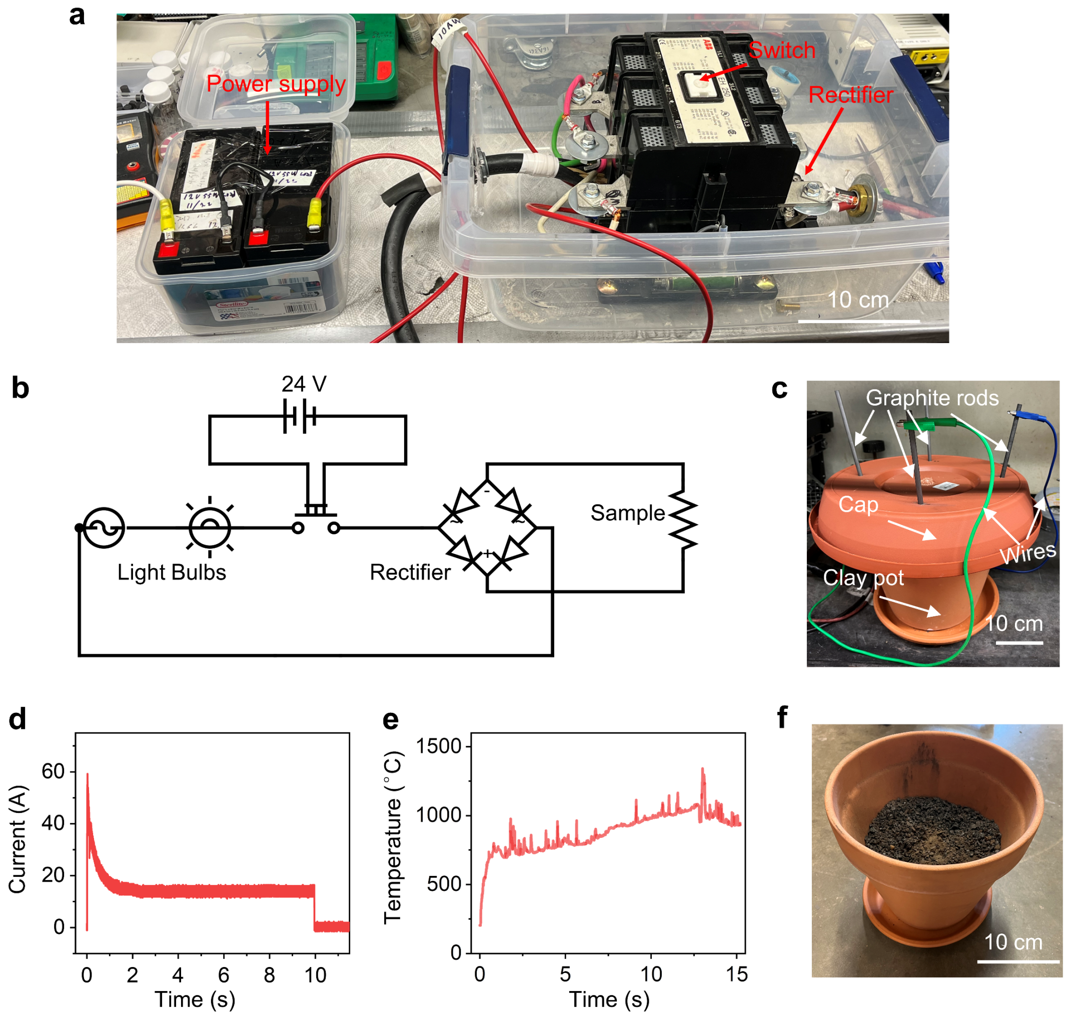

Supplementary Fig. 55. Scaling up for kilogram-scale soil remediation. a, Picture of the kilogram-scale REM equipment. It can convert commercial alternative current (AC) input to direct current (DC) output. **b,** Electrical diagram of the scale-up REM system. **c,** Picture of the reaction pot with a plastic cap. 2 kg of contaminated soil mixed with 500 g of metcoke. **d,** Current profile during the REM process. **e,** Real-time temperature curve of the large-scale sample recorded by an infrared thermometer. The temperature detection range of the thermometer is 200 - 1500 °C. **f,** Picture of REM soil mixed with metcoke.

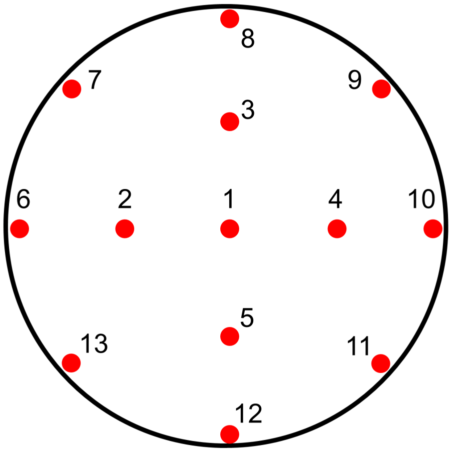

Supplementary Fig. 56. Soil sample collecting points in each plane for the kilogram-scale REM soil. 13 points were collected in each plane and 4 different depths (with an interval of 2 cm) were collected for the LC-MS tests and further calculations of PFOA removal efficiencies.

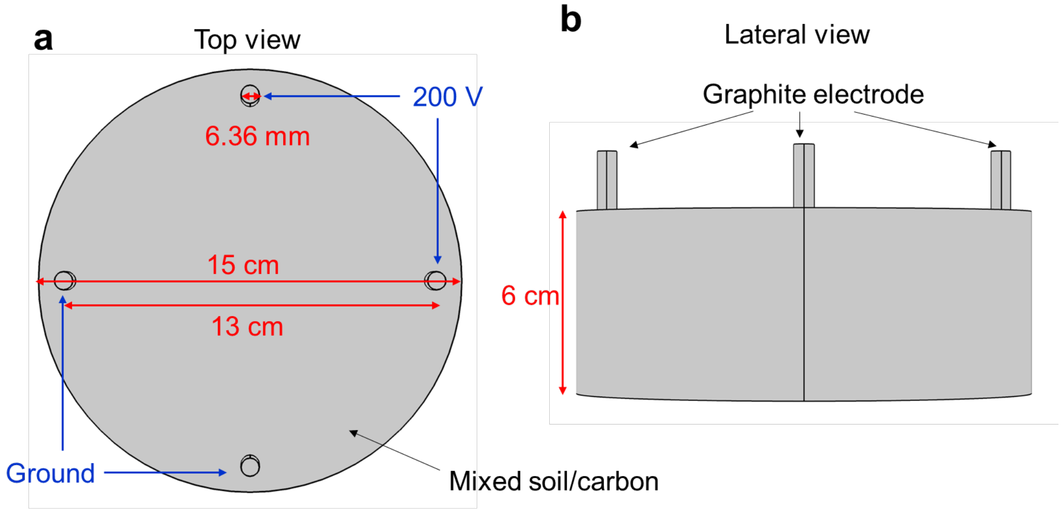

Supplementary Fig. 57. Geometry and boundary conditions for the simulation. a, Top view. **b,** Lateral view.

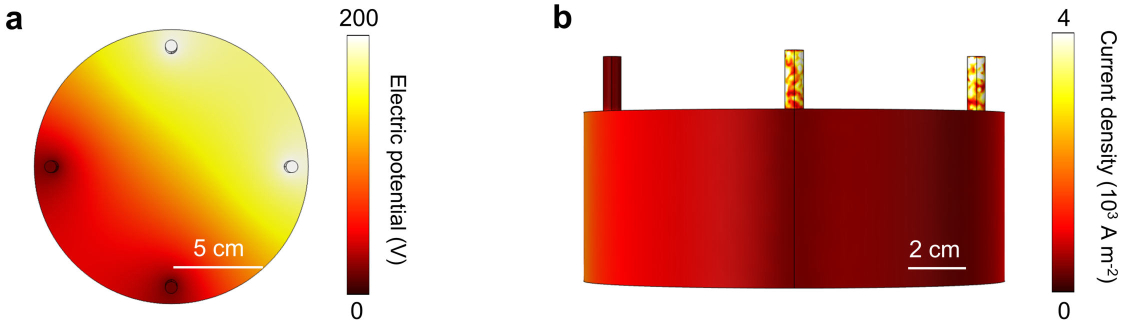

Supplementary Fig. 58. Simulated electric field and current density distribution during the kilogram-scale REM process. a, Simulated potential distribution. **b,** Simulated cross-section current density distribution.

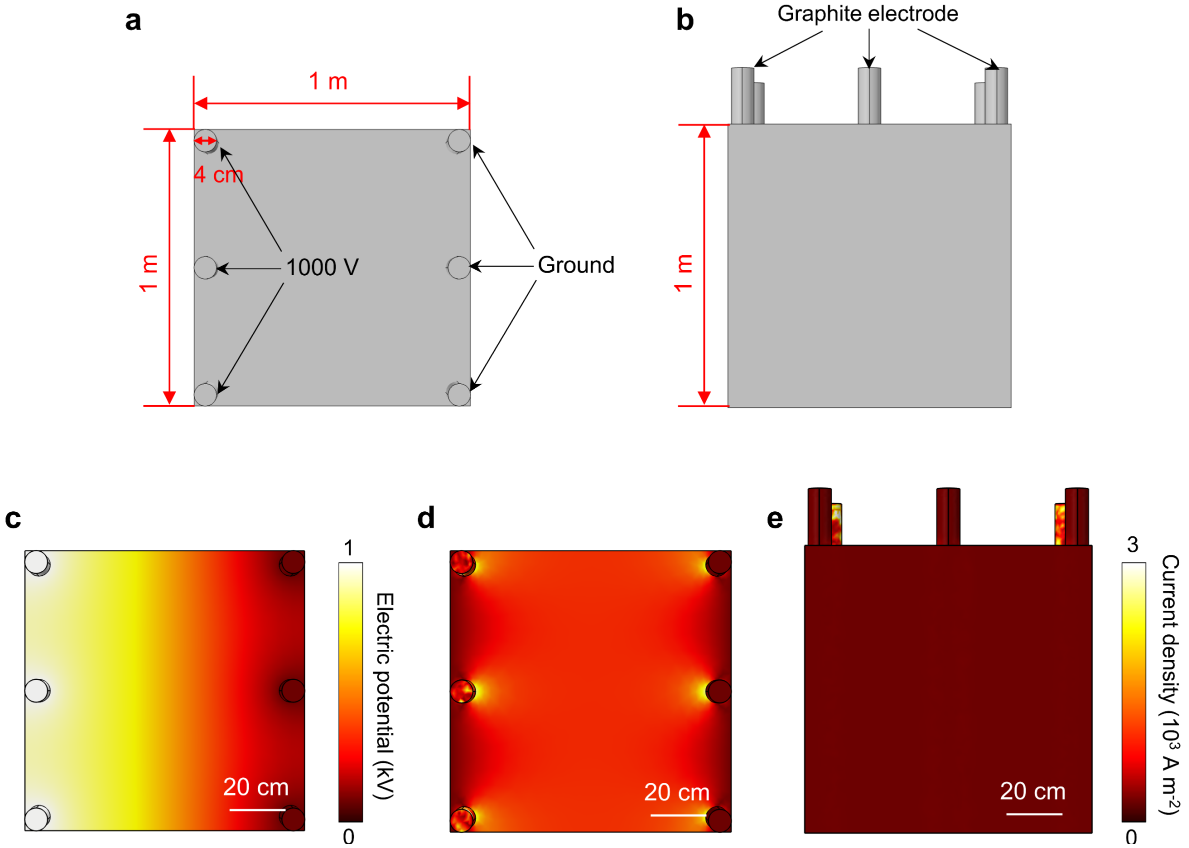

Supplementary Fig. 59. Electric simulations at a 1 m × 1 m × 1 m field with 1000 V input. a, Top view of the simulation geometry. **b,** Lateral view of the geometry simulation. **c,** Simulated

potential distribution. **d,** Simulated in-plane current density distribution. **e,** Simulated cross-section current density distribution.

To further demonstrate the scalability of REM at the field-scale level, we conducted the electric simulations of REM at a 1 m × 1 m × 1 m field with 1000 V input (Supplementary Fig. 59), where the current density in the central position is similar with that in the clay pot (Fig. 5b) of ~800 A m^{-2}. It indicates a comparable temperature of ~1000 °C can be achieved under such a voltage input. Meanwhile, the current density is uniform both in-plane and in-depth (Supplementary Fig. 59d,e), proving the homogeneous heating capability for field-scale soil remediation.

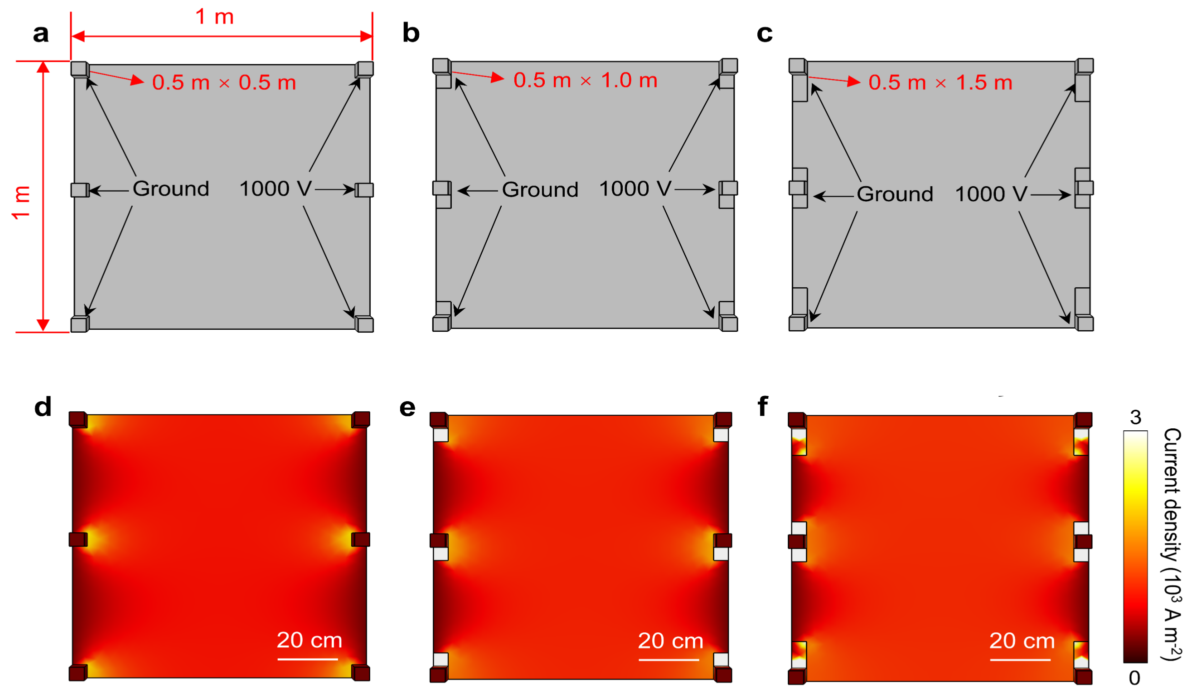

Supplementary Fig. 60. Simulated current density distribution with different electrode surface areas. a-c, Geometry and boundary conditions. **d-e,** Current density distribution.

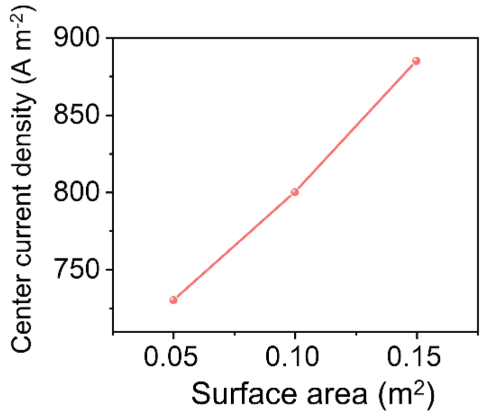

Supplementary Fig. 61. Current density at the center position versus different electrode surface area.

The current density distribution was simulated in an on-site REM with a volume of 1 m × 1 m × 1 m with different electrode areas (Supplementary Fig. 60). With the increase of each electrode

surface area from 0.25 to 0.75 m^2, the current density at the center position increases from 730 to 870 A m^2 (Supplementary Fig. 61), which indicates a higher REM temperature. Therefore, during REM, a higher electrode surface area can facilitate a higher REM temperature and thus a higher PFOA removal efficiency.

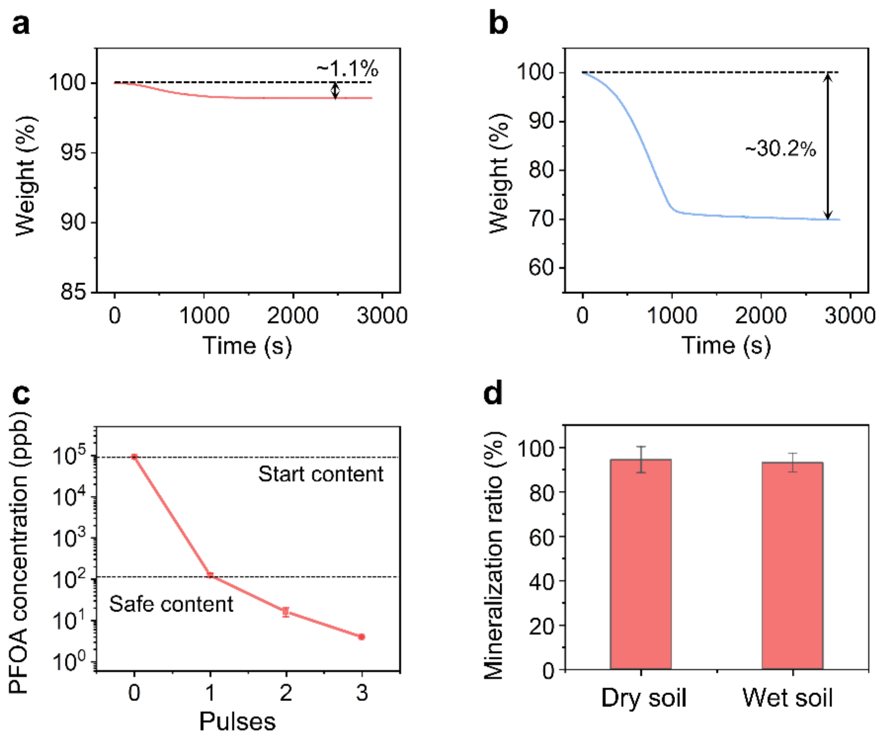

Supplementary Fig. 62. PFOA mineralization in wet soil. a, TGA curve of dry soil. **b,** TGA curve of wet soil. TGA is conducted in air with a heating rate of 5 °C min-1 and then kept at 110 °C for 30 min. **c,** Residual PFOA concentrations in soil after repetitive electric pulses, with an input voltage of 100 V and duration of 1 s each time. **d,** Comparison of mineralization ratios in dry soil and wet soil. The error bars in **c** and **d** denote standard deviations, where $N = 3$.

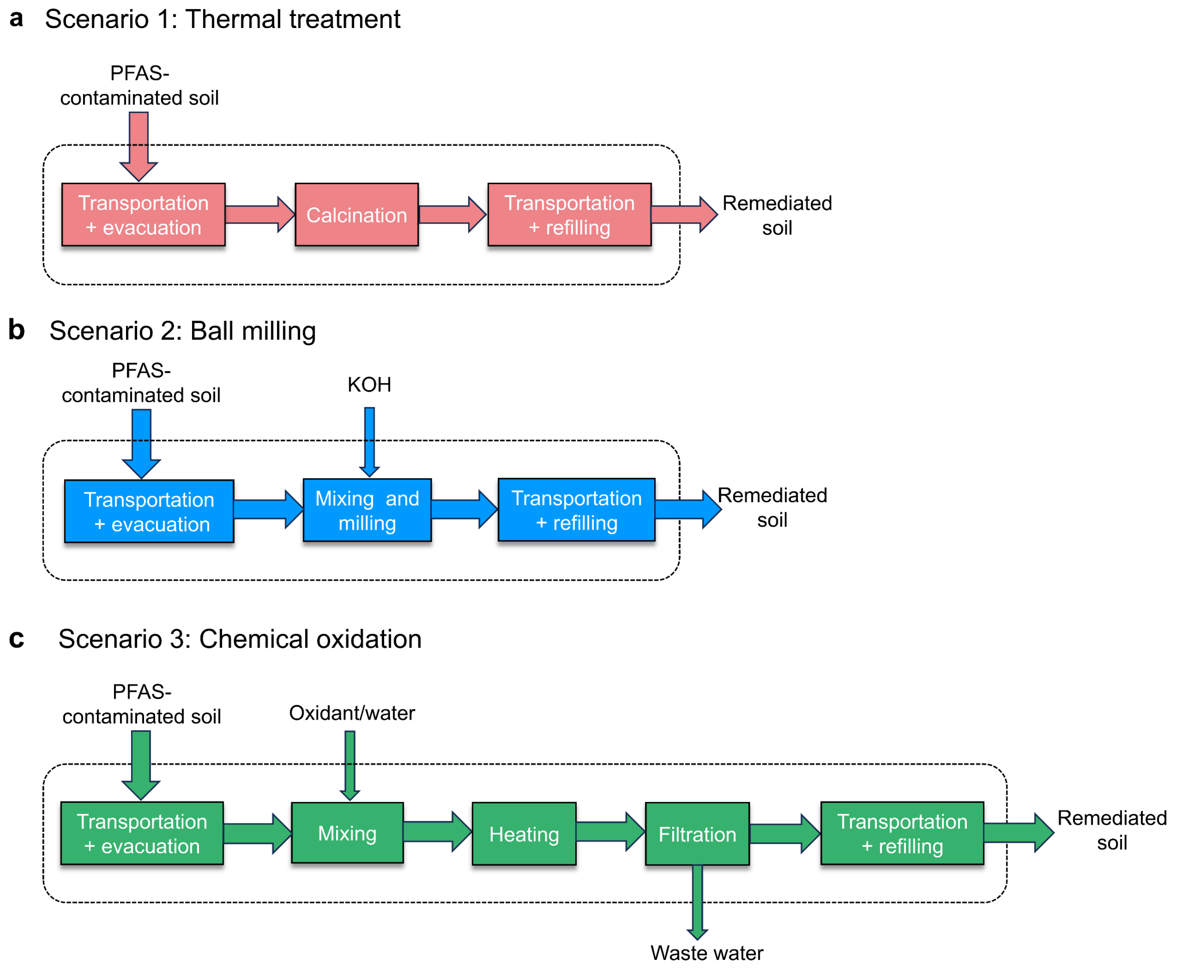

Supplementary Fig. 63. Flow chart representation and boundary conditions for different LCA scenarios. a, Scenario: Thermal treatment. **b,** Scenario: Chemical oxidation. **c,** Scenario: Ball milling.

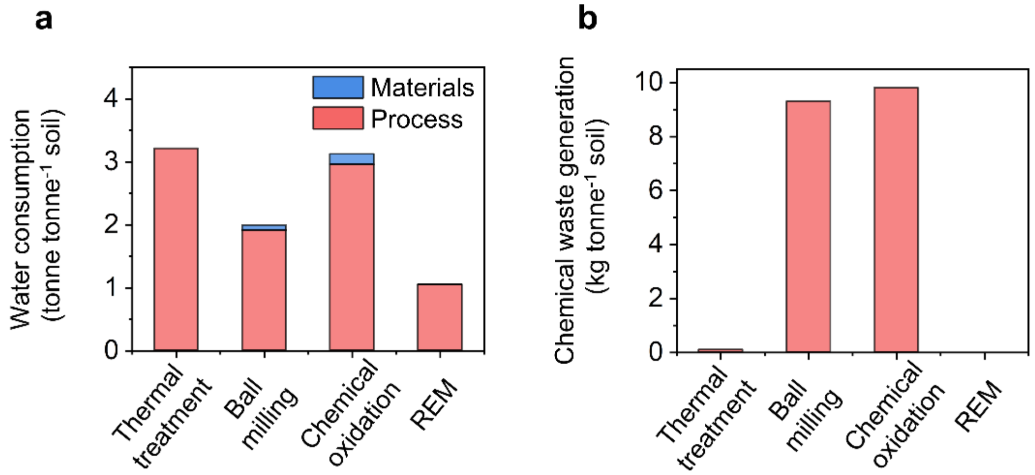

Supplementary Fig. 64. Environmental impact comparisons between different PFAS removal methods. a, Comparison of cumulative water consumption. **b,** Comparison of cumulative chemical waste generation.

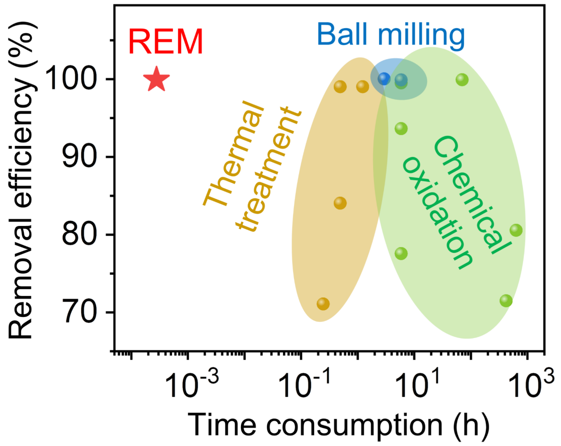

Supplementary Fig. 65. The comparison of treating time and PFAS removal efficiencies between REM and other methods, including thermal treatment[5,25], ball milling[7], and chemical oxidation[6,26].

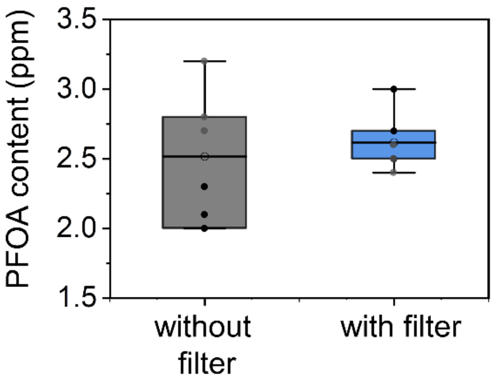

Supplementary Fig. 66. Boxplot with individual data points of PFOA contents extracted from soil with and without the PES filter. *N* = 6. The central line represents the median value. Box limits represent upper and lower quartiles. Whiskers represent the 5th and 95th percentiles.

Supplementary Table 1. Physical properties of different kinds of PFAS used in this work.

Precursors	Formula	Molecular weight ($g\ mol^{-1}$)	F mass ratio (%)	Mixed concentration in the soil (ppm)
PFOA	$C_8HF_{15}O_2$	414.1	68.8	146.5
PFOS	$C_{16}H_{20}F_{17}NO_3S$	629.4	51.3	223.2
PFH_xS	$C_6F_{13}O_3SK$	438.2	56.4	155.0
PFBS	$C_4F_9O_3SK$	338.2	50.6	119.2
PTFE	$-CF_2CF_2-$	/	76.0	105.2

Note: * PFOS, PFH_xS and PFBS here are the corresponding salt mentioned in the Methods section.
** The mixed PFOA, PFOS, PFH_xS and PFBS concentrations in the soil were tested by LC-MS.
*** The mixed PTFE concentration in the soil was tested by CIC.

Supplementary Table 2. Parameters for REM of soil.

Precursors	Mass Ratio	Mass (mg) **	Resistance (Ω)	Voltage (V)	Time (s)	Mass after REM (mg) ***
c-Soil(PFOA):biochar	2:1	300	3.5	40	1	271
c-Soil(PFOA):biochar	2:1	300	3.5	60	1	253
c-Soil(PFOA):biochar	2:1	300	3.5	80	1	245
c-Soil(PFOA):biochar	2:1	300	3.5	100	1	238
c-Soil(PFOA):biochar	2:1	300	3.5	120	1	227
c-Soil(PFOA):biochar	2:1	300	3.5	150	1	223
c-Soil(PFOA):biochar	1:1	300	2.5	100	1	221
c-Soil(PFOA):biochar	3:1	300	10.5	100	1	249
c-Soil(PFOA):biochar	4:1	300	18.5	100	1	282
c-Soil(wet, PFOA): biochar	2:1	300	4.5	100	1	192
c-Soil(PFOA): biochar (4 mm inner diameter tube)	2:1	108	2.1	60	1	76
c-Soil(PFOA):recycled biochar	2:1	300	3.5	100	1	247
c-Soil(PFOA):carbon black	2:1	300	1.0	100	1	241
c-Soil(PFOA):metcoke	2:1	302	1.5	100	1	236
c-Soil(PFOA):recycled metcoke	2:1	305	1.0	100	1	243

c-Soil(PFOA):metcoke	1:1	300	1.0	100	1	219
c-Soil(PFOA):metcoke	3:1	300	1.5	100	1	238
c-Soil(PFOA):metcoke	4:1	300	1.5	100	1	247
SiO_2(PFOA):metcoke	2:1	300	1.5	100	1	265
c-Soil(PFOA):flash graphene	2:1	305	1.0	100	1	249
c-Soil(PFOS):biochar	2:1	300	3.5	120	1	242
c-Soil(PFH_xS):biochar	2:1	300	3.5	120	1	248
c-Soil(PFBS):biochar	2:1	300	3.5	120	1	241
c-Soil(PTFE):biochar	2:1	300	3.5	80	1	252
c-Soil(PTFE):biochar	2:1	300	3.5	100	1	229
c-Soil(PTFE):biochar	2:1	300	3.5	120	1	212
c-Soil(PTFE):biochar	2:1	300	3.5	150	1	206

Note: *c-Soil is abbreviation of contaminated soil. **The total mass includes c-Soil and carbon additives; ***The total mass includes residual carbon additives.

Supplementary Table 3. Detecting limits of different PFAS characterization methods.

Characterization methods	Detecting limit
FT-IR	1 wt% (ref[27])
XRD	0.5 wt% (ref[28])
^{19}F-NMR	50 ppb (ref[29])
HPLC-DAD	500 ppb
QQQ LC-MS	0.1 ppb

Supplementary Table 4. Parameters for REM by mixing metal salt with PFOA*.

Metal salts	Mass (mg)**	PFAS type	Mass (mg)	Mass of metcoke (mg)	Resistance (Ω)	Mass after REM (mg)***
$CaCO_3$	137	PFOA	63	100	2.5	212
$CaCO_3$	116	PFOS	84	99	2.5	216
$CaCO_3$	128	PFH_xS	72	100	2.5	208
$CaCO_3$	123	PFBS	77	102	2.5	205
$CaCO_3$	140	PTFE	59	101	3.0	220
$MgCO_3$	131	PFOA	70	100	2.6	197
Na_2CO_3	139	PFOA	61	100	2.2	221

Note: * The input voltage was set as 100 V, REM time was set as 1 s and total mass for each REM is ~300 mg. **Metal counterion content is 1.2 mole equivalents compared with the F mole content in PFAS. ***The total mass includes residual carbon additives.

Supplementary Table 5. Metal-fluorine bond energy.

M-F type	Bond energy (kJ mol^{-1})
Na-F	477
Mg-F	463
K-F	489
Ca-F	529

Supplementary Table 6. pH and cation exchange capacity (CEC) of different soil samples.

Soil types	pH	CEC (cmol kg^{-1})
Raw soil	7.19 ± 0.02	15.25 ± 0.81
REM soil	7.58 ± 0.03	15.45 ± 1.04
Calcined soil	10.63 ± 0.08	4.08 ± 0.37

Supplementary Table 7. Soil nutrient concentrations.

Nutrients	Raw soil (ppm)	Calcinated soil (ppm)	REM soil (ppm) **	Biochar (ppm)
P	68 ± 1	50 ± 2	137 ± 14	328 ± 69
Mg	255 ± 20	139 ± 9	406 ± 9	1189 ± 92
K	280 ± 11	71 ± 3	423 ± 7	3232 ± 195
Ca	2605 ± 109	1281 ± 569	3230 ± 275	3942 ± 168
Mn	133 ± 3	265 ± 40	156 ± 17	265 ± 40
N*	16 ± 3	0.74 ± 0.08	17 ± 2	0.23 ± 0.05
Fe	311 ± 12	432 ± 138	294 ± 64	307 ± 64

Note: *The nutrient N refers to nitrate nitrogen; **REM soil tested here is the soil after removing the biochar inside.

Supplementary Table 8. Results from the generalized linear models testing for the main and interactive effects of soil types and culture time on springtail survival.

	**Survival ratio**	
Predictors	*CI*	*p*
(Intercept)	0.18 – 1.44	0.203
Soil Type [Raw soil]	0.29 – 4.83	0.819
Soil Type [REM soil]	0.46 – 6.26	0.430
Soil Type [PFOA soil]	0.00 – Inf.	0.997
Week [Week_2]	0.00 – Inf.	0.997
Week [Week_3]	0.00 – Inf.	0.997
Soil Type [Raw soil] × Week [Week_2]	0.00 – Inf.	0.997
Soil Type [REM soil] × Week [Week_2]	0.00 – Inf.	0.997
Soil Type [PFOA soil] × Week [Week_2]	0.00 – Inf.	0.998
Soil Type [Raw soil] × Week [Week_3]	0.00 – Inf.	1.000
Soil Type [REM soil] × Week [Week_3]	0.00 – Inf.	0.998
Soil Type [PFOA soil] × Week [Week_3]	0.00 – Inf.	0.998
Observations	84	

Supplementary Table 9. Results from the generalized linear models testing for the main and interactive effects of soil types and culture time on isopod survival.

	Survival ratio		
Predictors	*Odds Ratios*	*CI*	*p*
(Intercept)	7.00	1.25 – 130.85	0.069
Soil Type [Raw soil]	1.00	0.03 – 28.82	1.000
Soil Type [REM soil]	1.00	0.03 – 28.82	1.000
Soil Type [PFOA soil]	0.05	0.00 – 0.49	0.024
Week [Week_2]	0.43	0.02 – 5.61	0.529
Week [Week_3]	0.14	0.01 – 1.39	0.129
Soil Type [Raw soil] × Week [Week_2]	1.44	0.03 – 84.59	0.850
Soil Type [REM soil] × Week [Week_2]	1.44	0.03 – 84.59	0.850
Soil Type [PFOA soil] × Week [Week_2]	1.00	0.02 – 52.84	1.000
Soil Type [Raw soil] × Week [Week_3]	1.00	0.02 – 45.26	1.000
Soil Type [REM soil] × Week [Week_3]	1.00	0.02 – 45.26	1.000
Soil Type [PFOA soil] × Week [Week_3]	0.00	0.00 – Inf.	0.995
Observations	96		

Supplementary Table 10. Parameters of electric field simulation.

Parameters	Soil	Graphite electrode
Shape	Cyclinder	Cyclinder
Size	15 cm (diameter),<br>6 cm (height)	6.36 mm (diameter),<br>8 cm (height)
Electrical conductivity	0.83 S m^{-1}	2×10^5 S m^{-1}
Relative permittivity	4	18
Boundary condition	200 V input	

Supplementary Table 11. Materials flow for various scenarios.

Scenarios	Thermal treatment (tonne)	Chemical oxidation (tonne)	Ball milling (tonne)	REM by biochar (tonne)	REM by metcoke (tonne)
PFAS-contaminated soil	1	1	1	1	1
Biochar	0	0	0	0.075	0
Biochar pretreatment	0	0	0	0.075	0
Metcoke	0	0	0	0	0.035
Potassium permanganate	0	0.01	0	0	0
Water	0	10	0	0	0
Potassium hydroxide	0	0	0.0095	0	0
Excavation and transportation	1	1	1	0	0
Mixing	0	1.01	1.0095	1.5	1.5
Furnace heating	1	0	0	0	0
Water bath heating	0	11.01	0	0	0
Filtration	0	11.01	0	0	0
Ball milling	0	0	1.0095	0	0
Transportation and refilling	1	1	1	0	0
REM	0	0	0	1.5	1.5
Cyclone separation	0	0	0	1.5	0
Sieving	0	0	0	0	1.5

Note: *The material mass flows are normalized to treat 1 tonne of PFAS-contaminated soil. **PFAS concentration in the contaminated soil is set as 100 ppm. ***REM, rapid electrothermal mineralization.

Supplementary Table 12. Life cycle inventory.

Impact Category	Energy consumption (MJ)	GHG emission (kg)	Water consumption (kg)
Biochar	20025	5.04	4.7
Metcoke	28030	362.6	416.9
Potassium permanganate	19480	1891	8682
Potassium hydroxide	28000	1980	17221
Biochar pretreatment	360	46.8	241.2
Excavation and transportation	451.5	33.8	302.5
Mixing	9.43	1.23	6.3
Furnace heating	3874	504	2595.6
Water bath heating	1866	243	1260.2
Filtration	2.2	0.29	1.5
Ball milling	3456	449	2315.5
Transportation and refilling	466.6	35.7	312.6
REM	1000	130	670
Cyclone separation	6.75	19	4.5
Sieving	4.2	0.55	2.8

Note: *The material mass flows are normalized to treat 1 tonne of PFAS-contaminated soil. **PFAS concentration in the contaminated soil is set as 100 ppm. ***REM, rapid electrothermal mineralization. ****GHG, greenhouse gas.

Supplementary Table 13. Energy consumption for various scenarios.

Scenarios	Thermal treatment (MJ)	Chemical oxidation (MJ)	Ball milling (MJ)	REM by biochar (MJ)	REM by metcoke (kg)
Biochar	0	0	0	1501.9	0
Metcoke	0	0	0	0	981.05
Potassium permanganate	0	194.8	0	0	0
Potassium hydroxide	0	0	266	0	0
SUM of Materials	0	194.8	266	1501.9	981.05
Excavation and transportation	451.5	451.5	451.5	0	0
Biochar pretreatment	0	0	0	27	0
Mixing	0	9.43	9.44	14.15	14.15

Furnace heating	3874	0	0	0	0
Water bath heating	0	1866	0	0	0
Filtration	0	24.2	0	0	0
Ball milling	0	0	3460	0	0
Transportation and refilling	466.6	466.6	466.6	0	0
REM	0	0	0	1500	1500
Cyclone separation	0	0	0	10.13	0
Sieving	0	0	0	0	6.30
SUM of Process	4792.1	2817.7	4387.5	1551.3	1520.45
**SUM**	**4792**	**3013**	**4654**	**3053**	**2502**

Note: *The material mass flows are normalized to treat 1 tonne of PFAS-contaminated soil. **PFAS concentration in the contaminated soil is set as 100 ppm. ***REM, rapid electrothermal mineralization.

Supplementary Table 14. GHG emissions for various scenarios.

**Scenarios**	**Thermal treatment (kg)**	**Chemical oxidation (kg)**	**Ball milling (kg)**	**REM by biochar (kg)**	**REM by metcoke (kg)**
Biochar	0	0	0	0.38	0
Metcoke	0	0	0	0	12.69
Potassium permanganate	0	18.9	0	0	0
Potassium hydroxide	0	0	18.81	0	0
SUM of Materials	0	18.9	18.8	0.4	12.69
Excavation and transportation	33.79	33.79	33.79	0	0
Biochar pretreatment	0	0	0	3.51	0
Mixing	0	1.23	1.24	1.84	1.84
Furnace heating	504	0	0	0	0
Water bath heating	0	243	0	0	0
Filtration	0	3.19	0	0	0
Ball milling	0	0	453	0	0
Transportation and refilling	35.75	35.75	35.75	0	0
REM	0	0	0	195	195
Cyclone separation	0	0	0	1.32	0

Sieving	0	0	0	0	0.83
SUM of Process	573.5	317.0	523.8	201.7	197.7
**SUM**	**574**	**336**	**543**	**202**	**210**

Note: *The material mass flows are normalized to treat 1 tonne of PFAS-contaminated soil. **PFAS concentration in the contaminated soil is set as 100 ppm. ***REM, rapid electrothermal mineralization. ****GHG, greenhouse gas.

Supplementary Table 15. Water consumption for various scenarios.

**Scenarios**	**Thermal treatment (kg)**	**Chemical oxidation (kg)**	**Ball milling (kg)**	**REM by biochar (kg)**	**REM by biochar (kg)**
Biochar	0	0	0	0.35	0
Metcoke	0	0	0	0	145.9
Potassium permanganate	0	86.82	0	0	0
Potassium hydroxide	0	0	163.6	0	0
SUM of Materials	0	86.8	163.6	0.35	145.9
Excavation and transportation	302.5	302.5	302.5	0	0
Biochar pretreatment	0	0	0	27	0
Mixing	0	6.36	6.36	9.45	9.45
Furnace heating	2595.6	0	0	0	0
Water bath heating	0	1272.8	0	0	0
Filtration	0	16.52	0	0	0
Ball milling	0	0	2337.5	0	0
Transportation and refilling	312.6	312.6	312.6	0	0
REM	0	0	0	1005	1005
Cyclone separation	0	0	0	6.75	0
Sieving	0	0	0	0	4.22
SUM of Process	3210.7	1910.8	2959	1048.2	1018.67
**SUM**	**3211**	**1998**	**3123**	**1049**	**1165**

Note: *The material mass flows are normalized to treat 1 tonne of PFAS-contaminated soil. **PFAS concentration in the contaminated soil is set as 100 ppm. ***REM, rapid electrothermal mineralization.

Supplementary Table 16. Materials and energy cost inventory.

Scenarios	Materials cost ($)	Energy cost ($)
Biochar	1400	0
Metcoke	150	0
Potassium permanganate	37700	0
Potassium hydroxide	31600	0
Water	1.085	0
Excavation and transportation	0	26.50
Biochar pretreatment	0	5.87
Mixing	0	0.15
Furnace heating	0	63.17
Water bath heating	0	30.43
Filtration	0	0.036
Ball milling	0	56.36
Transportation and refilling	0	27.39
REM	0	16.31
Cyclone separation	0	0.17
Sieving	0	0.068

Note: *The material mass flows are normalized to treat 1 tonne of PFAS-contaminated soil. **PFAS concentration in the contaminated soil is set as 100 ppm. ***REM, rapid electrothermal mineralization. ****The consumed energy is assumed to be from electricity, and the industrial price of electrical energy in Texas, USA is $0.0587 kWh[1].

Supplementary Table 17. Cost evaluation of various scenarios.

Scenarios	Thermal treatment ($)	Chemical oxidation ($)	Ball milling ($)	REM by biochar ($)	REM by metcoke ($)
Biochar	0	0	0	105	0
Metcoke	0	0	0	0	5.25
Potassium permanganate	0	377	0	0	0
Potassium hydroxide	0	0	300.2	0	0
Water	0	10.85	0	0	0
SUM of Materials	0	388	300	105	5.25
Transportation and refilling	26.50	26.50	26.50	0	0
Biochar pretreatment	0	0	0	0.44	0.23
Mixing	0	0.15	0.15	0.23	0
Furnace heating	63.17	0	0	0	0
Water bath heating	0	30.43	0	0	0

Filtration	0	0.39	0	0	0
Ball milling	0	0	56.42	0	0
Transportation and refilling	27.39	27.39	27.39	0	0
REM	0	0	0	24.46	24.46
Cyclone separation	0	0	0	0.17	0
Sieving	0	0	0	0	0.10
SUM of Process	117	85	110	25	24.89
**SUM**	**117**	**473**	**411**	**130**	**30**

Note: *REM, rapid electrothermal mineralization. **PFOA, perfluorooctanoic acid; PFOS, perfluorooctane sulfonates. ***Material price: industrial water ($1.085 per tonne, ref[18]), $KMnO_4$ ($37.7 per kg, ref[19]), KOH ($31.6 per kg, ref[20]) biochar ($1400 per tonne, ref[2]) and metcoke ($150 per tonne, ref[3]). ****The consumed amount of biochar during REM process is calculated by subscribing the recycled mass from the input mass.

Supplementary References

1. https://www.eia.gov/electricity/monthly/epm_table_grapher.php?t=epmt_5_6_a, accessed Sept. 5th, 2023.
2. https://fedcoseeds.com/ogs/raw-biochar-8348, accessed Sept. 5th, 2023.
3. https://www.alibaba.com/product-detail/10-80mm-metallurgical-coke-price-carbon_1600716599553.html?s=p, accessed Sept. 5th, 2023.
4. ISO, 2006. Life cycle assessment-requirements and guidelines (ISO 14044:2006).
5. Sörengård, M., Lindh, A. & Ahrens, L. Thermal desorption as a high removal remediation technique for soils contaminated with per-and polyfluoroalkyl substances (PFASs). *PloS one* **15**, e0234476 (2020).
6. Liu, C. S., Shih, K. & Wang, F. Oxidative decomposition of perfluorooctanesulfonate in water by permanganate. *Sep. Purif. Technol.* **87**, 95-100 (2012).
7. Zhang, K. et al. Destruction of perfluorooctane sulfonate (PFOS) and perfluorooctanoic acid (PFOA) by ball milling. *Environ. Sci. Technol.* **47**, 6471-6477 (2013).

8. https://greet.es.anl.gov/, accessed Sept. 5th, 2023.
9. https://www.lfatabletpresses.com/vh-powder-mixer, accessed Sept. 5th, 2023.
10. Cappuyns, V. & Kessen, B. Evaluation of the environmental impact of brownfield remediation options: Comparison of two life cycle assessment-based evaluation tools. *Environmental technology* **33**, 2447-2459 (2012).
11. Nanaki, E. A. & Koroneos, C. J. Comparative LCA of the use of biodiesel, diesel and gasoline for transportation. *J Clean. Prod.* **20**, 14-19 (2012).
12. https://www.grainger.com/product/38l778, accessed Sept. 5th, 2023.
13. http://www.alibaba.com/product-detail/widely-used-water-cooled-ball-mill_1600296928133.html, accessed Sept. 5th, 2023.
14. https://www.alibaba.com/product-detail/industrial-collector-fine-powder-cyclone-air_1600482316874.html, accessed Sept. 5th, 2023.
15. Nabavi-Pelesaraei, A., Bayat, R., Hosseinzadeh-Bandbafha, H., Afrasyabi, H. & Chau, K.-w. Modeling of energy consumption and environmental life cycle assessment for incineration and landfill systems of municipal solid waste management-A case study in Tehran Metropolis of Iran. *J Clean. Prod.* **148**, 427-440 (2017).
16. https://www.alibaba.com/product-detail/industrial-collector-fine-powder-cyclone-air_1600482316874.html, accessed Sept. 5th, 2023.
17. Deng, B. et al. Heavy metal removal from coal fly ash for low carbon footprint cement. *Commun. Eng.* **2**, 13 (2023).
18. https://www.cityofbryan.net/commercialindustrial-metering-rates/, accessed Sept. 5th, 2023.
19. https://www.laballey.com/products/potassium-permanganate-lab?variant=39891260473499, accessed Sept. 5th, 2023.

20. https://www.laballey.com/products/potassium-hydroxide-flake-lab?variant=7219047628859, accessed Sept. 5th, 2023.

21. Deng, B. et al. Urban mining by flash Joule heating. *Nat. Commun.* **12**, 5794 (2021).

22. Luong, D. X. et al. Gram-scale bottom-up flash graphene synthesis. *Nature* **577**, 647-651 (2020).

23. Lopez-Capel, E., Sohi, S. P., Gaunt, J. L. & Manning, D. A. Use of thermogravimetry–differential scanning calorimetry to characterize modelable soil organic matter fractions. *Soil Sci. Soc. Am. J.* **69**, 136-140 (2005).

24. Huang, P.-J. et al. Reusable functionalized hydrogel sorbents for removing long-and short-chain perfluoroalkyl acids (PFAAs) and genx from aqueous solution. *ACS omega* **3**, 17447-17455 (2018).

25. Endpoint, Bench-scale VEG research & development study: Implementation memorandum for ex-situ thermal desorption of perfluoroalkyl compounds (PFCs) in soils. (2017).

26. Bolan, N. et al. Remediation of poly-and perfluoroalkyl substances (PFAS) contaminated soils–to mobilize or to immobilize or to degrade? *J. Hazard. Mater.* **401**, 123892 (2021).

27. Gorrochategui, E., Lacorte, S., Tauler, R. & Martin, F. L. Perfluoroalkylated substance effects in *Xenopus laevis* A6 kidney epithelial cells determined by ATR-FTIR spectroscopy and chemometric analysis. *Chem. Res. Toxicol.* **29**, 924-932 (2016).

28. Hillier, S. Quantitative analysis of clay and other minerals in sandstones by X-ray powder diffraction (XRPD). *Clay mineral cements in sandstones*, 213-251 (1999).

29. Camdzic, D. et al. Quantitation of total pfas including trifluoroacetic acid with fluorine nuclear magnetic resonance spectroscopy. *Anal. Chem.* **95**, 5484-5488 (2023).
