# Supplementary material for: Electrothermal mineralization of per- and polyfluoroalkyl substances for soil remediation
Source: Nat Commun. 2024 Jul 20;15:6117. doi: 10.1038/s41467-024-49809-6 (PMC11271446; doi:10.1038/s41467-024-49809-6)
Supplement: Supplementary file 3 — Description of Additional Supplementary Files [file 41467_2024_49809_MOESM3_ESM.pdf]

Description of Additional Supplementary Files

File Name: Supplementary Data 1
Description: The PFAS atomic coordinates and structure configurations are provided in Supplementary Data 1.
